# Antioxidant capacity of phenolics in *Camellia nitidissima* Chi flowers and their identification by HPLC Triple TOF MS/MS

**DOI:** 10.1371/journal.pone.0195508

**Published:** 2018-04-10

**Authors:** Rui Yang, Ying Guan, Weixin Wang, Hongjuan Chen, Zhaochun He, Ai-Qun Jia

**Affiliations:** 1 Key Laboratory of Tropical Biological Resources of Ministry Education, State Key Laboratory of Marine Resource Utilization in South China Sea, Hainan University, Haikou, China; 2 School of Environmental and Biological Engineering, Nanjing University of Science and Technology, Nanjing, China; 3 Inspection and Pattern Evaluation Department, Suzhou Institute of Measurement and Testing, Suzhou, China; 4 State Key Laboratory of Pharmaceutical Biotechnology, Nanjing University, Nanjing, China; Mizoram University, INDIA

## Abstract

*Camellia nitidissima* Chi (CNC) is a valuable medicinal and edible plant in China. In this study, CNC flowers were extracted with 95% ethanol, then partitioned into dichloromethane, ethyl acetate, *n*-butanol, and water fractions, with the antioxidant capacity of flavonoids and other phytochemicals in CNC flowers investigated for the first time. Results showed that the ethyl acetate fraction exhibited the strongest antioxidant capacity and highest total phenolic content (TPC) compared with the other fractions. Furthermore, in the ethyl acetate fraction, the 50% effective concentrations (EC_50_) of ABTS^+^ and DPPH radical scavenging activities were 64.24 ± 1.80 and 78.80 ± 0.34 μg/mL, respectively, and the ferric reducing antioxidant power (FRAP) was 801.49 ± 2.30 μM FeSO_4_ at 1,000 μg/mL. Pearson’s correlation coefficients and principal component analyses (PCA) for the TPC and antioxidant capacity of the five fractions indicated that the phenolic compounds were the major antioxidant constituents in the flowers. To exploit the antioxidants in CNC flowers, 21 phenolic compounds in the ethanolic extract fraction were identified by HPLC Triple TOF MS/MS, next, 12 flavonoids were isolated and elucidated, of which compounds **1**–**5** showed potent antioxidant capacity. In addition, the potential structure-activity relationship among these 12 flavonoids showed that (1) the *o*-catechol group in the B-ring was primarily responsible for the antioxidant capacity of flavonoids and (2) steric hindrance, produced by glycosides and other groups, could reduce the antioxidant capacity of the flavonoids.

## Introduction

Reactive oxygen species (ROS) are a vital factor in many human diseases such as neurodegeneration, arthritis, coronary heart disease, emphysema, cancer, and aging [1, 2]. Thus, investigating antioxidants to reduce the incidence of disease due to oxidative damage is essential. Recently clarified side-effects of synthetic antioxidants have pushed research and development into new safe and effective natural antioxidants from fruits, vegetables, and herbs [1, 3, 4]. Recently, phytochemicals are regarded as reducing agents that can scavenge free radicals or metal ion chelators, thereby reducing oxidative stress in the human body and exerting a beneficial effect on health [4–6].

Phenolic compounds are a major plant constituent, and are distributed widely in many fruits, vegetables, and herbs. Phenolic compounds have attracted considerable attention because of their biological activities, including antioxidant, antiviral, anti-inflammatory, and anticancer [7]. Flavonoids are a large group of phenolic compounds in plants, and are divided based on structural differentiation into flavanols, flavanones, flavonols, isoflavones, flavones, and anthocyanins. Flavonoids and flavonoid analogues have a long history of antioxidant use, and thus play important roles in human health [7].

*Camellia nitidissima* Chi (CNC) belongs to the *Camellia* genus (Theaceae family). As a rare species in the world, CNC often referred to as the “panda of the plant kingdom” in China [8]. It is a medicinal and edible plant, which can regulate serum lipids and suppress hepatocellular carcinoma proliferation, as well as exhibit anti-inflammation capability [9]. In addition, CNC leaves have been shown to demonstrate antioxidant activity [8], although compound diversity in different teas shows different antioxidant activities [10]. To date, however, the antioxidant activity and antioxidant composition of CNC flowers have not yet been investigated. Thus, the objective of this study was to evaluate the antioxidant capacity of the phytochemicals in CNC flowers.

## Materials and methods

### Instruments and chemicals

The nuclear magnetic resonance data (^1^H-NMR and ^13^C-NMR) were measured on a Bruker AV-500 (Bruker Inc., Germany). Mass spectrometry (MS) was performed on Agilent 1100 Series LC-MSD-Trap/SL and Thermo TSQ Quantum LC/MS spectrometers (USA). Silica gel (100–200 mesh, 200–300 mesh) and thin-layer chromatography (TLC) were purchased from Qingdao Marine Chemical Factory (Qingdao, China). Sephadex LH-20 (GE Healthcare Bio-Sciences AB, Uppsala, Sweden), C_18_ (YMC, Japan), and RP-18 F_254_ plates (0.25 mm, Merck, Germany) were prepared. We purchased 2, 2-diphenyl-1-picrylhydrazyl (DPPH), 2, 4, 6-tris (2-pyridyl)-S-triazine (TPTZ), 2, 2-azinobis (3-ethylbenzothiazoline-6-sulfonic acid) diammonium salt (ABTS), and ascorbic acid (Vc) from Sigma-Aldrich Chemical Co. (St. Louis, MO, USA). Gallic acid and Folin-Ciocalteu’s reagent were purchased from Merck (Darmstadt, Germany). HPLC grade methanol was obtained from Tedia (Fairfield, USA). All other chemicals were of analytical grade and purchased from Shuangling Chemical Reagent Co. (Nanjing, China). The CNC flowers were collected in 2015 from Guangxi Province, China, and a voucher specimen (JHCF-001) was kept in our lab. After the flowers were air-dried, they were powdered (ca. 40 mesh) and stored at 4 ^o^C until use.

### Extraction of phytochemicals and compounds

The phytochemical extractions were prepared following our previous study [11], with minor modifications. The CNC flowers (6 kg) were refluxed with 95% ethanol for 3 times (3, 2, and 1 h, respectively), then combined and evaporated in a rotary evaporator at 45 ^o^C to yield the ethanolic extract. Ethanolic extracts (1.2 kg), suspended in 6 L water, were extracted three times with dichloromethane (6 L). Dichloromethane extracts were combined and the solvent was evaporated to yield the dichloromethane fraction (52 g). Next, the left water suspension was extracted with ethyl acetate and *n*-butanol for three times sequently, then the ethyl acetate (256 g) and *n*-butanol fractions (560 g) were obtained. The resuidual water phase was dried at 50 ^o^C to yield the water fraction (300 g). The dichloromethane fraction was subjected to silica gel column chromatography eluted with a gradient system (dichloromethane-methanol 1:0, 49:1, 25:1, 15:1, 9:1, 5:1, 0:1) to yield 4 subfractions based on TLC analysis. Subfraction-3 was repeatedly subjected to silica gel column chromatography, Sephadex LH-20 (dichloromethane-methanol 1:1), and C_18_ (methanol-water 2:8→10:0) to yield compound **12** (3.25 g). Similarly, the ethyl acetate fraction was subjected to silica gel column chromatography to obtain 17 subfractions. With the use of silica gel column chromatography, Sephadex LH-20 (dichloromethane-methanol 1:1) and C_18_ (methanol-water 2:8→10:0) columns, compound **11** (2.16 g) was isolated from subfraction-9, compounds **1** (2.57 g), **3** (78 mg), and **5** (62 mg) were isolated from subfraction-10, compounds **6** (10 mg) and **7** (25 mg) were isolated from subfraction-11, compounds **4** (118 mg) and **8** (78.5 mg) were isolated from subfraction-13, and compounds **2** (32 mg), **9** (512 mg), and **10** (19 mg) were isolated from subfraction-14.

### Determination of total phenolic content (TPC)

Total phenolic content (TPC) was determined by the Folin-Ciocalteu method [11]. Briefly, 100 μL of the five fractions at suitable concentrations, 1.15 mL of deionized water, 0.25 mL of Folin-Ciocalteu’s reagent, and 1 mL of 7.5% sodium carbonate solution were mixed. Samples were placed in darkness at room temperature for 1 h after vortexing. Absorbance was read at 760 nm using a spectrophotometer (Thermo Electron Corp., Waltham, MA, USA). Results were expressed as milligrams of gallic acid equivalent per gram of material (mg GAE/g material).

### Ethanolic extract assay using HPLC Triple TOF MS/MS

The ethanolic extract assay was carried out by HPLC Triple TOF MS/MS according to previous research [12], with minor modifications. Briefly, the ethanolic extract was analyzed on a Shimadzu HPLC equipped with a diode array detector, and a Welch Ultimate XB-C18 column (100 mm × 2.1 mm i.d., 3 μm; Welch Materials, Inc., Shanghai, China). Mobile phase A was 0.1% formic acid of water and mobile phase B was 0.1% formic acid of methanol. The linear gradient was: 0–1 min, 5–5% B; 1–30 min, 5–70% B; 30–35 min, 70–90% B; 35–40 min, 90–90% B; 40–40.1 min, 90–5% B; 40.1–45 min, 5–5% B. The flow rate was 0.4 mL/min and the injection volume was 10 μL. A Triple TOF 4600 system (AB SCIEX, CA) with electrospray ionization was operated at negative modes. The following parameter settings were used: ion spray voltage, 4.5 kV; ion source heater, 550 ^o^C; curtain gas, 25 psi; ion source gas 1, 55 psi; and ion source gas 2, 55 psi. Mass spectra were scanned from *m/z* 100 to 1500. The collision energy was swept from 30 to 60 eV for MS/MS analysis.

### Measurements of antioxidant activity

#### ABTS radical cation scavenging activity assay

The ABTS radical cation scavenging activity of the samples were examined in accordance with an earlier study [13], with minor modifications. To generate ABTS^+^, ABTS (7 mM) and potassium persulfate (2.45 mM) were incubated in the dark at room temperature for 16 h. The freshly prepared ABTS^+^ solution was diluted with ethanol to obtain an absorbance at 734 nm of 0.70 ± 0.02 before the test. Approximately, 100 μL of each sample at different concentrations was added to 400 μL of the ABTS^+^ solution and adequately mixed. The concentrations of the fractions were 0, 25, 50, 100, 200, 400, 600, 800, and 1,000 μg/mL, and of the compounds were 0, 2.5, 5, 12.5, 25, 50, 100, 200, and 400 μg/mL. The reactive mixture was placed in the dark at room temperature for 6 min. Absorbance was then recorded at 734 nm. The ABTS^+^ scavenging activity was calculated as follows, ABTS^+^ scavenging activity (%) = [1 –A_sample_/A_control_] × 100, where A_sample_ is the absorbance in the presence of the sample and A_control_ is the absorbance of the blank without the test sample. The ABTS^+^ scavenging activity of Vc was assayed for positive control.

#### Determination of DPPH radical scavenging activity

The DPPH radical scavenging activity of the samples was determined following [14], with minor modifications. Briefly, 400 μL of each sample at different concentrations was added to 400 μL of DPPH solution (0.4 mM). The concentrations of the fractions were 0, 25, 75, 100, 125, 150, 200, and 400 μg/mL, and of the compounds were 0, 2.5, 5, 12.5, 25, 50, 100, 200, and 400 μg/mL. The mixture was shaken immediately and incubated in the dark at room temperature for 30 min. Absorbance was recorded at 517 nm. The DPPH radical scavenging activity was calculated as follows: DPPH radical scavenging activity (%) = [1 –A_sample_/A_control_] × 100, where A_sample_ is the absorbance in the presence of the sample and A_control_ is the absorbance of the blank without the fraction. The DPPH radical scavenging activity of Vc was used as a positive control.

#### Evaluation of ferric reducing antioxidant power (FRAP)

The ferric reducing antioxidant power (FRAP) of the samples was evaluated according to previous research [15], with some modifications. Briefly, 10 mL of TPTZ solution (10 mM, in 40 mM HCl), 100 mL of acetate buffer (0.3 M, pH 3.6), and 10 mL of ferric chloride (20 mM) were mixed to prepare fresh FRAP working solution, which was warmed at 37 °C prior to testing. We added 200 μL of each sample at different concentrations to the FRAP solution (1 mL), with the mixture then placed in a 37 °C water bath for 20 min. The concentrations of the fractions were 0, 25, 100, 200, 400, 600, 800, and 1,000 μg/mL, and of the compounds were 0, 2.5, 5, 12.5, 25, 50, 100, 200, and 400 μg/mL. Absorbance was read at 593 nm. Different concentrations (10–1,600 μg/mL) of ferrous sulfate were used to prepare a standard curve. Results were expressed as μM Fe (II). FRAP of Vc was also used as a positive control.

#### Statistical analyses

All experiments were independently conducted in triplicate, and experimental results were expressed as means ± standard deviations or average. One-way analysis of variance (ANOVA) and Duncan’s multiple range tests were performed using SPSS version 17.0 (SPSS Inc., Chicago, IL, USA) software. Statistical significance was determined at *p* < 0.05. Interpolation from linear regression analysis was used to obtain the EC_50_. In order to interpret the relationships between antioxidant activity and total phenolic contents, two-tailed Pearson’s correlation coefficient analysis and principal component analysis (PCA) were conducted using SPSS version 17.0 (SPSS Inc., Chicago, IL, USA) software.

## Results and discussion

### Total phenolic content (TPC)

**Fig 1:**
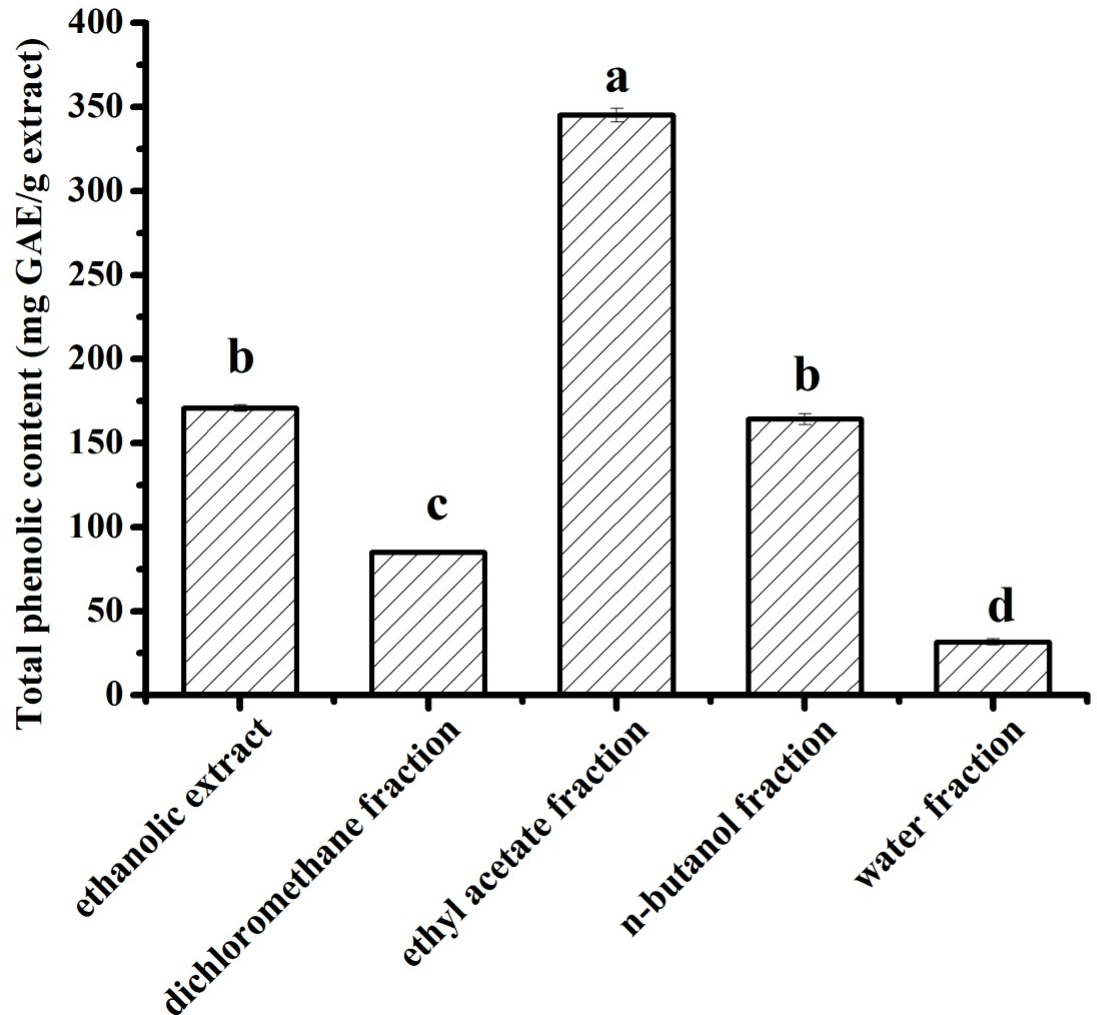
Total phenolic content in the five fractions of *C*. *nitidissima* Chi flowers. Each value is expressed as mean ± SD (n = 3). Different letters showed significant differences from each other.

*C*. *nitidissima* Chi flowers are used as a popular tea in China. As the main phytochemicals of tea, phenolic compounds play an important role in biological activities [10]. As shown in **Fig 1**, the CNC flower fractions contained many phenolic compounds. The TPC of the ethyl acetate fraction was highest and that of the water fraction was lowest (345.14 ± 4.05 and 31.69 ± 1.75 mg GAE/g, respectively) among all fractions. The TPC of the *n*-butanol fraction was 164.19 ± 3.18 mg GAE/g, similar to that of the ethanolic extract (170.74 ± 1.99 mg GAE/g). The TPC of the dichloromethane fraction (85.02 ± 0.88 mg GAE/g) was significantly lower than that of the ethyl acetate fraction, *n*-butanol fraction, and ethanolic extract [11]. These results indicate that phenolic compounds in this species can be solubilized in medium polar solvents, such as water-saturated ethyl acetate [12, 16].

### Evaluation of compounds 1–12

**Fig 2:**
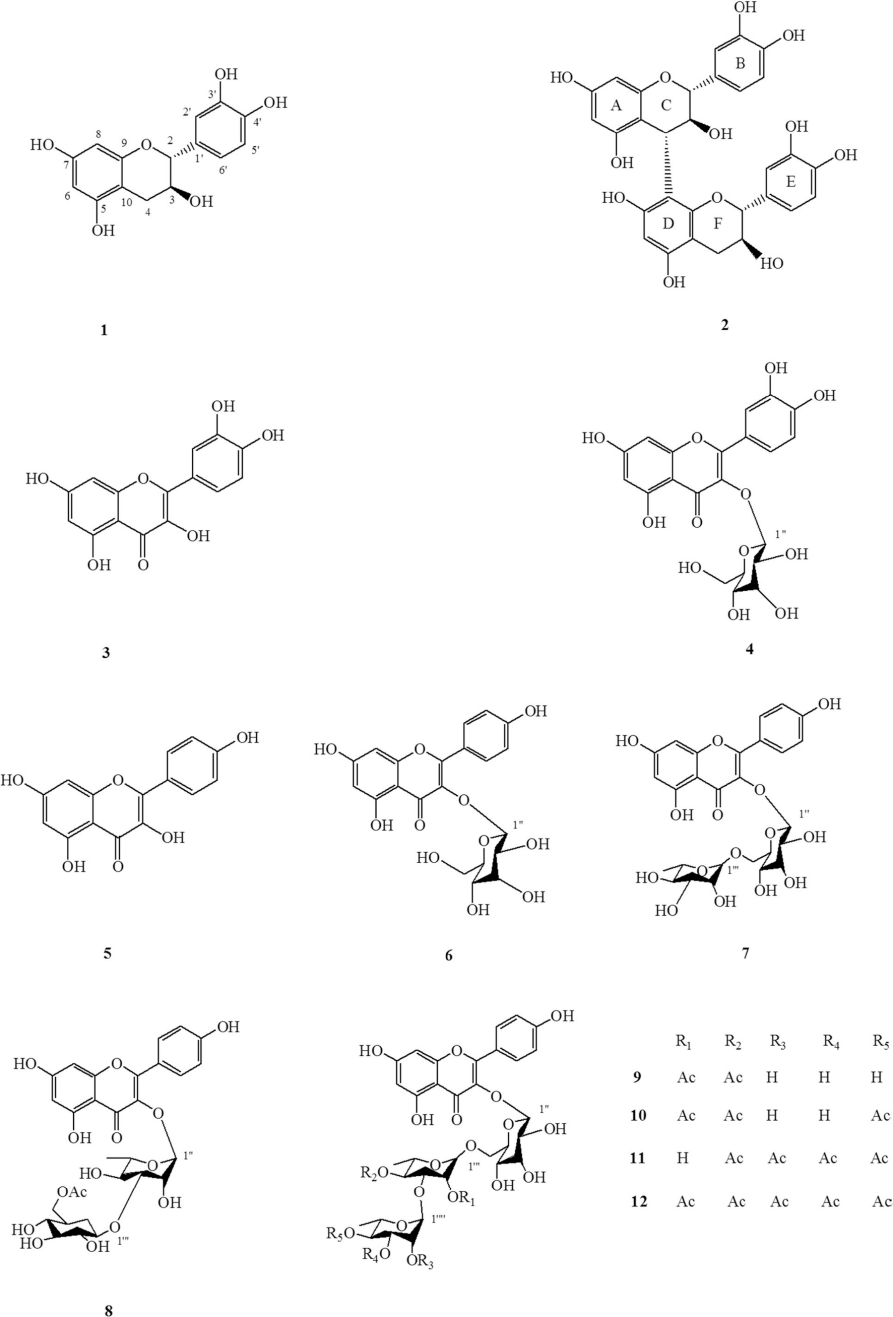
Chemical structures of compounds 1–12.

Twelve flavonoids were isolated and elucidated from the CNC flowers (**Fig 2**). All 9 flavonoids besides **1**, **11**, and **12** were identified in CNC flowers for the first time.

Compound **1**, Catechin. Yellow amorphous powder. C_15_H_14_O_6_. ESI-MS, *m/z* 289.01 [M-H]^-^. ^1^H-NMR (500 MHz, CD_3_OD) δ 6.84 (1H, d, *J* = 1.5 Hz, H-2’), 6.77 (1H, d, *J* = 8.0 Hz, H-5’), 6.73 (1H, dd, *J* = 2.0, 8.0 Hz, H-6’), 5.93 (1H, d, *J* = 2.5 Hz, H-8), 5.86 (1H, d, *J* = 2.5 Hz, H-6), 4.57 (1H, d, *J* = 7.5 Hz, H-2), 4.00 (1H, m, H-3), 2.87 (1H, dd, *J* = 5.5, 16.5 Hz, H-4), 2.53 (1H, dd, *J* = 8.0, 16.0 Hz, H-4). ^13^C-NMR (125 MHz, CD_3_OD), shown in **Table 1**[11].

**Table 1:** ^13^C-NMR data of compounds 1–12 isolated from *C*. *nitidissima* Chi flowers (δ in ppm and all in CD_3_OD at 125 MHz).

	1	2	3	4	5	6	7	8	9	10	11	12
2	81.4	82.6 (C), 81.1 (F)	146.3	158.6	148.1	158.8	157.1	158.9	158.2	158.7	158.3	158.5
3	67.4	72.3 (C), 67.5 (F)	137.4	135.8	137.2	135.6	134.1	135.2	135.0	135.8	135.2	135.0
4	27.1	37.2 (C), 27.4 (F)	177.4	179.5	177.4	179.5	178.1	179.2	179.1	179.7	179.1	179.5
5	156.4	155.7 (A), 153.5 (D)	162.6	163.2	162.6	163.1	161.6	162.8	162.7	163.1	162.8	163.1
6	95.0	96.0 (A), 95.5 (D)	99.4	100.1	99.4	100.5	100.0	99.9	99.9	102.1	99.8	100.0
7	156.6	155.7 (A), 154.5 (D)	165.7	166.3	165.6	167.6	164.3	165.8	165.6	166.3	165.7	167.0
8	94.2	94.7 (A), 106.8 (D)	94.6	94.9	94.6	95.2	94.9	94.8	94.8	95.1	94.9	94.9
9	155.5	157.8 (A), 154.3 (D)	158.3	159.1	158.3	159.1	158.0	158.3	158.7	159.5	159.2	158.9
10	99.5	105.8 (A), 100.9 (D)	104.7	105.8	104.7	105.5	105.6	105.5	105.5	105.8	105.6	105.7
1’	130.8	130.5 (B), 131.2 (E)	124.3	123.4	123.9	123.0	121.4	122.6	122.6	122.8	122.7	122.9
2’	113.9	114.8 (B), 114.3 (E)	116.1	116.1	130.8	132.4	131.0	132.2	132.1	132.6	132.1	132.3
3’	148.8	144.7 (B), 144.4 (E)	148.1	149.0	116.4	116.2	114.7	116.1	116.1	116.3	115.8	116.3
4’	148.8	144.1 (B), 144.1 (E)	148.9	150.0	160.6	161.7	160.1	161.4	161.2	161.8	161.2	161.6
5’	114.8	114.7 (B), 115.1 (E)	116.4	117.7	116.4	116.2	114.7	116.1	116.1	116.3	115.8	116.3
6’	118.8	119.3 (B), 118.5 (E)	121.8	123.2	130.8	132.4	131.0	132.2	132.1	132.6	132.1	132.3
1”				104.5		104.4	104.6	104.0	103.6	105.5	104.2	103.3
2”				75.9		75.9	75.8	75.7	75.6	75.2	75.0	75.4
3”				78.5		78.2	76.7	70.2	76.6	75.6	77.1	76.7
4”				71.3		71.5	70.0	68.2	70.8	71.1	70.5	70.1
5”				78.2		78.5	74.4	75.3	77.8	80.6	77.8	78.1
6”				62.9		62.8	67.2	17.6	67.7	64.5	68.5	67.6
1”‘							102.4	102.1	98.7	101.1	99.4	99.0
2”‘							70.9	72.0	73.0	71.9	72.6	72.7
AcO-C(2”‘)									171.8 20.8	171.8 20.8		171.9 20.8
3”‘							70.7	78.0	75.9	73.2	76.3	76.0
4”‘							72.5	71.0	73.8	72.5	73.5	73.9
AcO-C(4”‘)									171.9 20.8	172.0 20.9	171.7 20.5	172.0 20.8
5”‘							68.3	76.7	67.3	67.9	67.9	67.3
6”‘							16.5	67.4	17.3	17.8	17.4	17.3
AcO-C(6”‘)								172.8 21.1				
1”“									103.5	102.6	100.3	100.0
2”“									72.2	71.2	71.4	71.2
AcO-C(2”“)											171.7 20.4	171.9 20.9
3”“									71.9	70.4	70.7	71.1
AcO-C(3”“)											171.5 20.6	171.6 21.1
4”“									73.5	73.0	71.8	72.1
AcO-C(4”“)										172.0 20.8	171.5 20.5	172.3 20.7
5”“									70.5	70.2	69.7	68.5
6”“									17.8	18.0	17.5	17.7

Compound **2**, Catechin-4 *α*, 8-catechin. Yellow amorphous powder. C_30_H_26_O_12_. ESI-MS, *m/z* 577.00 [M-H]^-^. ^1^H-NMR (500 MHz, CD_3_OD) δ 6.96 (1H, d, *J* = 1.8Hz, 5’E-H), 6.83 (1H, m, 5’B-H), 6.74 (1H, d, *J* = 1.8 Hz, 2’B-H), 6.69 (1H, dd, *J* = 8.2, 2.4 Hz, 6’B-H), 6.60 (1H, d, *J* = 1.8 Hz, 2’E-H), 6.47 (1H, dd, *J* = 8.2, 1.8 Hz, 6’E-H), 6.07 (1H, s, 6D-H), 5.89 (1H, d, *J* = 2.4 Hz, 6A-H), 5.79 (1H, d, *J* = 2.4 Hz, 8A-H), 4.55 (1H, d, *J* = 7.4 Hz, 2F-H), 4.42 (1H, d, *J* = 8.0 Hz, 2C-H), 4.37(1H, d, *J* = 9.7 Hz, 4C-H), 4.26 (1H, m, 3C-H), 3.81 (1H, m, 3F-H), 2.78 (1H, dd, *J =* 5.6, 16 Hz, 4F*α*-H), 2.51 (1H, m, 4F*β*-H). ^13^C-NMR (125 MHz, CD_3_OD), shown in **Table 1**[17].

Compound **3**, Quercetin. Yellow amorphous powder. C_15_H_10_O_7_. ESI-MS, *m/z* 300.95 [M-H]^-^. ^1^H-NMR (500 MHz, CD_3_OD) δ 7.72 (1H, d, *J* = 2.1 Hz, H-2’), 7.63 (1H, dd, *J* = 8.5, 2.1 Hz, H-6’), 6.89 (1H, d, *J* = 8.5 Hz, H-5’), 6.37 (1H, d, *J* = 2.1 Hz, H-8), 6.17 (1H, d, *J* = 2.1 Hz, H-6). ^13^C-NMR (125 MHz, CD_3_OD), shown in **Table 1**[18].

Compound **4**, Isoquercitrin. Yellow amorphous powder. C_21_H_20_O_12_. ESI-MS, *m/z* 462.96 [M-H]^-^. ^1^H-NMR (500 MHz, CD_3_OD) δ 7.71 (1H, d, *J* = 1.9 Hz, H-2’), 7.60 (1H, dd, *J* = 1.9, 8.5 Hz, H-6’), 6.87 (1H, d, *J* = 8.5 Hz, H-5’), 6.38 (1H, d, *J* = 1.7 Hz, H-8), 6.19 (1H, d, *J* = 1.7 Hz, H-6), 5.27 (1H, d, *J* = 8.6 Hz, H-1”), 3.21–3.51 (4H, m, H-2”, 3”, 4”, 5”), 3.73 (1H, dd, *J* = 11.9, 2.1 Hz, H-6a”), 3.60 (1H, dd, *J* = 11.9, 5.4 Hz, H-6b”). ^13^C-NMR (125 MHz, CD_3_OD), shown in **Table 1**[19].

Compound **5**, Kaempferol. Yellow amorphous powder. C_15_H_10_O_6_. ESI-MS, *m/z* 284.97 [M-H]^-^. ^1^H-NMR (500 MHz, CD_3_OD) δ 8.05 (2H, d, *J* = 8.9 Hz, H-2’, 6’), 6.89 (2H, d, *J* = 8.9 Hz, H-3’, 5’), 6.35 (1H, d, *J* = 2.0 Hz, H-8), 6.15 (1H, d, *J* = 2.0 Hz, H-6). ^13^C-NMR (125 MHz, CD_3_OD), shown in **Table 1**[18].

Compound **6**, Kaempferol 3-*O*-*β*-D-glucopyranosyl. Yellow amorphous powder. C_21_H_20_O_11_. ESI-MS, *m/z* 446.97 [M-H]^-^. ^1^H-NMR (500 MHz, CD_3_OD) δ 8.06 (2H, d, *J* = 8.6 Hz,H-2’, 6’), 6.89 (2H, d, *J* = 8.6 Hz, H-3’, 5’), 6.43(1H, s, H-8), 6.19 (1H, s, H-6), 5.25 (1H, d, *J* = 7.2 Hz, H-1”), 3.71 (1H, dd, *J* = 11.9, 2.0 Hz, H-6a”), 3.55 (1H, dd, *J* = 12.0, 5.5 Hz, H-6b”), 3.29–3.46 (4H, m, H-2”, 3”, 4”, 5”). ^13^C-NMR (125 MHz, CD_3_OD), shown in **Table 1**[20].

Compound **7**, Kaempferol-3-O-*β*-D-rutinoside. Yellow amorphous powder. C_27_H_30_O_15_. ESI-MS, *m/z* 593.00 [M-H]^-^. ^1^H-NMR (500 MHz, CD_3_OD) δ 8.07 (2H, d, *J* = 8.9 Hz, H-2’, 6’), 6.90 (2H, d, *J* = 8.9 Hz, H-3’, 5’), 6.40 (1H, d, *J* = 2.1 Hz, H-8), 6.21 (1H, d, *J* = 2.1 Hz, H-6), 5.14 (1H, d, *J* = 7.4 Hz, H-1”), 4.52 (1H, d, *J* = 1.1 Hz, H-1”‘), 3.24–3.82 (10H, m, H-2”, 3”, 4”, 5”, 6a”, 6b”, 2”‘, 3”‘, 4”‘, 5”‘), 1.13 (3H, d, *J* = 6.2 Hz, H-6”‘). ^13^C-NMR (125 MHz, CD_3_OD), shown in **Table 1**[21].

Compound **8**, Multiflorin C. Yellow amorphous powder. C_29_H_32_O_16_. ESI-MS, *m/z* 635.01 [M-H]^-^. ^1^H-NMR (500 MHz, CD_3_OD) δ 8.06 (2H, d, *J* = 8.9 Hz, H-2’, 6’), 6.91 (2H, d, *J* = 8.9 Hz, H-3’, 5’), 6.40 (1H, d, *J* = 2.1 Hz, H-8), 6.21 (1H, d, *J* = 2.1 Hz, H-6), 5.33 (1H, d, *J* = 7.3 Hz, H-1”), 4.82 (1H, m, H-4”‘), 4.57 (1H, s, H-1”‘), 3.82 (1H, br. d, *J* = 11.0, Ha-6”‘), 3.73 (1H, m, H-4”), 3.69 (1H, dd, *J* = 3.4, 10.0, H-3”), 3.38–3.59 (6H, m, Hb-6”‘,5”‘, 3”‘, 2”‘, 2”, 5”), 2.00 (3H, s, H_Me_-6”‘), 0.87 (3H, d, *J* = 6.6 Hz, H-6”). ^13^C-NMR (125 MHz, CD_3_OD), shown in **Table 1**[22].

Compound **9**, Kaempferol 3-*O*-[*α*-L-Rhamnopyranosyl-(1→3)-2,4-di-*O*-acetyl-*α*-L-rhamnopyranosyl-(1→6)]-*β*-D-Glucopyranoside. Yellow amorphous powder. C_37_H_44_O_21_. ESI-MS, *m/z* 823.23 [M-H]^-^. ^1^H-NMR (500 MHz, CD_3_OD) δ 8.04 (2H, d, *J* = 8.9 Hz, H-2’, 6’), 6.93 (2H, d, *J* = 8.9 Hz, H-3’, 5’), 6.40 (1H, d, *J* = 2.1 Hz, H-8), 6.22 (1H, d, *J* = 2.1 Hz, H-6), 5.41 (1H, d, *J* = 7.5 Hz, H-1”), 5.06 (1H, m, H-2”), 4.84 (1H, m, H-4”‘), 4.71 (1H, s, H-1”“), 4.62 (1H, s, H-1”‘), 3.93 (1H, dd, *J* = 3.5, 10.0, H-3”‘), 3.81 (1H, br. d, *J* = 10.4, Ha-6”), 3.80 (1H, dd, *J* = 1.5, 11.0 Hz, H-3”‘), 3.58–3.67 (5H, m, Hb-6”,5”‘, 2”“, 3”“,5”“), 3.40–3.53 (4H, m, H-3”,4”, 2”, 5”), 3.33 (1H, s, H-4”“), 2.07 (1H, s, H_Me_-2”‘), 1.96 (1H, s, H_Me_-4”‘), 1.19 (3H, d, *J* = 6.3, H-6”“), 0.83 (3H, d, *J* = 6.3 Hz, H-6”‘). ^13^C-NMR (125 MHz, CD_3_OD), shown in **Table 1**[23].

Compound **10**, Kaempferol 3-*O*-[4-*O*-Acetyl-*α*-L-rhamnopyranosyl-(1→3)-2,4-di-*O*-acetyl-*α*-L-rhamnopyranosyl-(1→6)]-*β*-D-glucopyranoside. Yellow amorphous powder. C_39_H_46_O_22_. ESI-MS, *m/z* 865.08 [M-H]^-^. ^1^H-NMR (500 MHz, CD_3_OD) δ 8.11 (2H, d, *J* = 8.9 Hz, H-2’, 6’), 6.89 (2H, d, *J* = 8.9 Hz, H-3’, 5’), 6.39 (1H, m, H-8), 6.21 (1H, d, *J* = 1.8 Hz, H-6), 5.37 (1H, m, H-1”), 5.09 (1H, d, *J* = 7.8 Hz, H-2”‘), 4.95–4.99 (2H, m, H-4”‘, 4”“), 4.52 (1H, br. s, H-1”“), 4.03 (1H, dd, *J* = 6.5, 9.8, H-3”‘), 3.65–3.74 (5H, m, H-5”‘, 2”“, 3”“, 5”“, 6”), 3.46–3.56 (5H, m, H-3”, 4”, 2”, 5”, 6”), 2.12 (3H, s, H_Me_-4”“), 2.05 (3H, s, H_Me_-2”‘), 1.94 (3H, s, H_Me_-4”‘), 1.18 (3H, d, *J* = 6.2, H-4”“), 1.04 (3H, d, *J* = 6.3, H-6”‘). ^13^C-NMR (125 MHz, CD_3_OD), shown in **Table 1**[23].

Compound **11**, Kaempferol 3-*O*-[2,3,4-Tri-*O*-acetyl-*α*-L-rhamnopyranosyl-(1→3)-4-*O*-acetyl-*α*-L-rhamnopyranosyl-(1→6)]-*β*-D-glucopyranoside. Yellow amorphous powder. C_41_H_48_O_23_. ESI-MS, *m/z* 907.10 [M-H]^-^. ^1^H-NMR (500 MHz, CD_3_OD) *δ* 8.04 (2H, d, *J* = 9.0 Hz, H-2’, 6’), 6.86 (2H, d, *J* = 9.0 Hz, H-3’, 5’), 6.38 (1H, d, *J* = 2.0 Hz, H-8), 6.21 (1H, d, *J* = 2.0 Hz, H-6), 5.28 (1H, dd, *J* = 1.5, 3.0 Hz, H-1”), 5.15 (1H, m, H-3”“), 5.02–5.08 (3H, m, H-4”‘, 4”“, 2”“), 4.95 (1H, d, *J* = 8.5 Hz, H-1”“), 4.49 (1H, s, H-1”‘), 3.81–3.92 (3H, m, H-6”, 2”‘, 5”“), 3.75 (1H, dd, *J* = 3.5, 8.5 Hz, H-3”‘), 3.35–3.50 (6H, m, H-3”, 4”, 5”, 2”, 6”, 5”‘), 2.13 (3H, s, H_Me_-3”“), 2.08 (3H, s, H_Me_-4”“), 2.06 (3H, s, H_Me_-2”“), 1.93 (3H, s, H_Me_-4”‘), 1.09 (3H, d, *J* = 5.0 Hz, H-6”“), 1.00 (3H, d, *J* = 5.0 Hz, H-6”‘). ^13^C-NMR (125 MHz, CD_3_OD), shown in **Table 1**[23].

Compound **12**, Kaempferol 3-*O*-[2,3,4-Tri-*O*-acetyl-*α*-L-rhamnopyranosyl-(1→3)-2,4-di-*O*-acetyl-*α*-L-rhamnopyranosyl-(1→6)]-*β*-D-glucopyranoside. Yellow amorphous powder. C_43_H_50_O_24_. ESI-MS, *m/z* 949.16 [M-H]^-^. ^1^H-NMR (500 MHz, CD_3_OD) *δ* 8.00 (2H, d, *J* = 9.0 Hz, H- 2’, 6’), 6.89 (2H, d, *J* = 9.0 Hz, H-3’, 5’), 6.38 (1H, d, *J* = 2.0 Hz, H-8), 6.21 (1H, d, *J* = 2.0 Hz, H-6), 5.48 (1H, d, *J* = 7.5 Hz, H-1”), 5.11 (1H, m, H-3”“), 5.00–5.06 (3H, m, H-4”‘, 4”“, 2”“), 4.94 (1H, m, H-1”“), 4.68 (1H, s, H-1”‘), 4.60 (1H, s, H-2”‘), 3.92–3.95 (2H, m, H-6”, 5”“), 3.80 (1H, dd, *J* = 1.5, 11.0 Hz, H-3”‘), 3.35–3.50 (6H, m, H-3”, 4”, 5”, 2”, 6”, 5”‘), 2.17 (3H, s, H_Me_-3”“), 2.12 (3H, s, H_Me_-4”“), 2.05 (3H, s, H_Me_-2”‘), 1.96 (3H, s, H_Me_-2”“), 1.95 (3H, s, H_Me_-4”‘), 1.13 (3H, d, *J* = 6.5, H-6”“), 0.77 (3H, d, *J* = 6.5 Hz, H-6”‘). ^13^C-NMR (125 MHz, CD_3_OD), shown in **Table 1**[23].

### Ethanolic extract analyses by HPLC Triple TOF MS/MS

**Fig 3:**
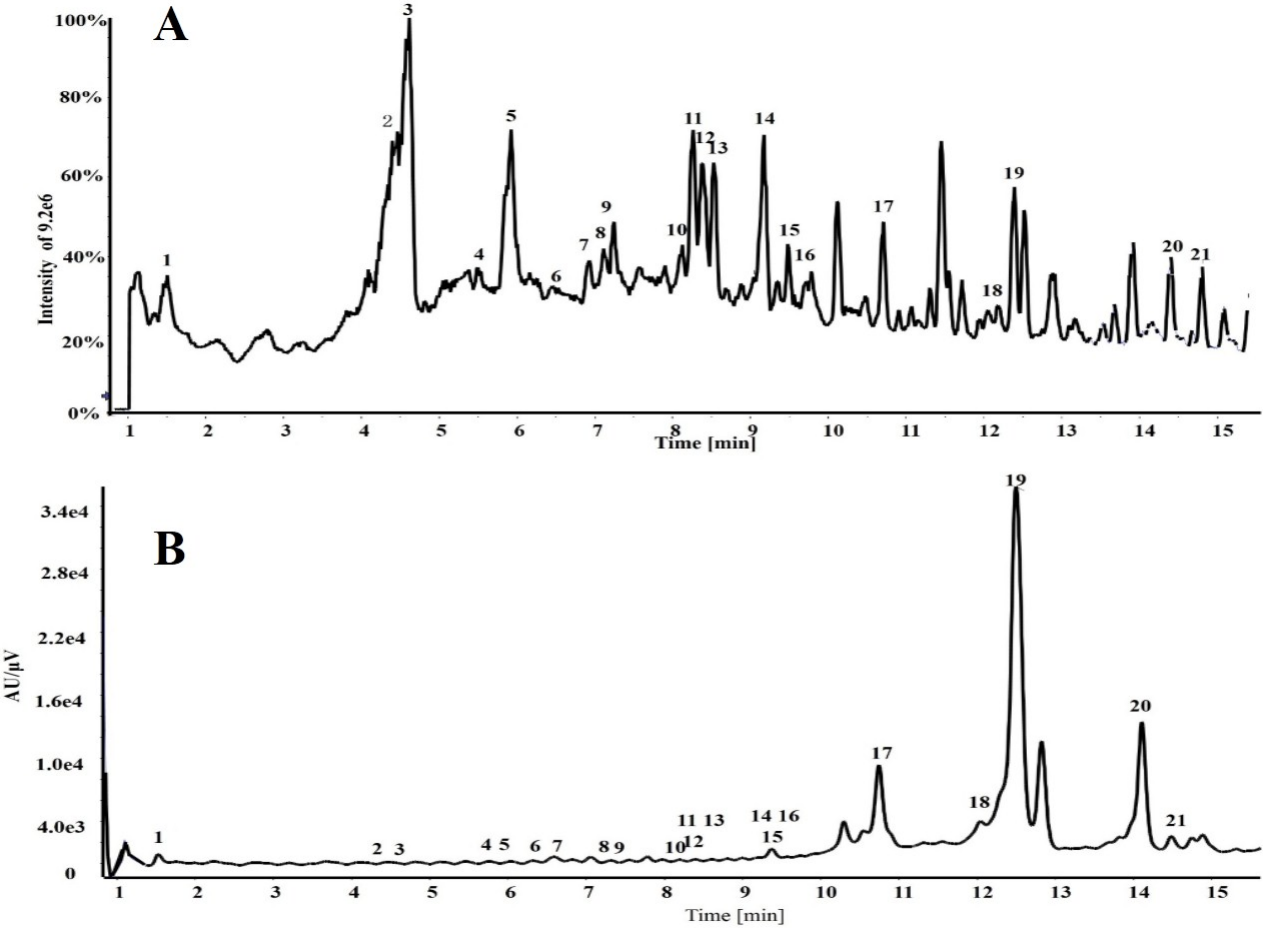
Total ion chromatogram of the *C*. *nitidissima* Chi flowers ethanolic extract (A); HPLC chromatogram of *Camellia nitidissima* Chi flower ethanolic extract by 360 nm detection (B).

Twenty-one phenolic compounds in the ethanolic extract of the CNC flower were identified (**Table 2**) by HPLC Triple TOF MS/MS analysis (**Fig 3**, **S1 Fig**).

**Table 2:** Mass spectrometric data of phenolic compounds identified in the ethanolic extract from *C*. *nitidissima* Chi flowers using HPLC Triple TOF MS/MS.

Peak	RT/min	Molecular formula	Tentative identification	Calculated [M-H]^-^	Measured [M-H]^-^	Error/ppm	MS/MS
1	1.56	C_7_H_6_O_5_	Gallic acid	169.01425	169.01424	0	125
2	4.29	C_30_H_26_O_12_	(*Epi*)catechin dimer	577.13515	577.13467	-0.8	451, 425, 407, 289
3	4.60	C_15_H_14_O_6_	Catechin	289.07176	289.07139	-1.3	245, 205, 203, 137
4	5.91	C_15_H_14_O_6_	*Epi*catechin	289.07176	289.07139	-1.3	245, 205, 203, 137
5	5.99	C_22_H_18_O_11_	Gallocatechin-gallate	457.07764	457.07721	-0.9	305, 169
6	6.49	C_37_H_30_O_16_	Procyanidin-gallate	729.14611	729.1455	-0.8	577, 559, 441, 407
7	6.89	C_33_H_40_O_21_	Quercetin-glucosyl-rhamnosyl-glucoside	771.19893	771.19835	-0.8	609, 463, 301
8	7.31	C_26_H_28_O_14_	Apigenin-pentosyl-glucoside	563.14063	563.1396	-0.1	545, 503, 473, 443, 383, 353
9	7.36	C_21_H_20_O_13_	Myricitrin-glucoside	479.08311	479.08279	-0.7	317, 316
10	8.12	C_33_H_40_O_20_	Kaempferol-glucosyl-rhamnosyl-glucoside	755.20402	755.20343	-0.8	593, 447, 285
11	8.14	C_22_H_18_O_10_	(*Epi*)catechin -gallate	441.08272	441.08213	-1.3	289, 169
12	8.21	C_21_H_20_O_10_	Vitexin	431.09837	431.09781	-1.3	311, 341
13	8.50	C_21_H_20_O_12_	Isoquercitrin	463.0882	463.08756	-1.4	301
14	9.12	C_21_H_20_O_11_	Kaempferol-galactoside	447.09329	447.09275	-1.2	285
15	9.16	C_27_H_30_O_15_	Kaempferol-rutinoside	593.15119	593.15081	-0.6	447, 285
16	9.49	C_21_H_20_O_11_	Kaempferol-glucoside	447.09329	447.09275	-1.2	285
17	10.43	C_17_H_24_O_9_	Syringin	371.14024	371.13898	-3	417, 209
18	12.17	C_15_H_10_O_7_	Quercetin	301.03538	301.03457	-2.7	273, 255, 179, 151
19	12.49	C_27_H_30_O_16_	Rutin	609.14611	609.1443	-1.8	301, 447
20	14.15	C_15_H_10_O_6_	Kaempferol	285.04046	285.03996	-1.8	239, 229, 211, 187
21	14.21	C_16_H_12_O_7_	Pollenitin	315.05103	315.05133	1.0	201, 229

The extract ion chromatogram at *m/z* 289.0712 showed two peaks at RT 4.60 and 5.91 min. These two peaks showed fragments at *m/z* 245, 205, 203, and 137 (**Table 2**), corresponding to the loss of CO_2_, C_4_H_4_O_2_, C_4_H_6_O_2_, and C_8_H_8_O_3_, respectively. The loss of C_4_H_4_O_2_ and C_4_H_6_O_2_ was due to the cleavage of the A ring in flavan-3-ol and the loss of C_8_H_8_O_3_ was through retro-Diels-Alder (RDA) fission [24]. Thus, the peak at 4.60 min was assigned to catechin and at 5.91 min was assigned to (*epi*)catechin [25, 26]. The extract ion chromatogram at *m/z* 577.1352 showed a peak at RT 4.29 min, which produced fragments at *m/z* 451, 425, 407, and 289 (**Table 2**) consistent with the loss of C_6_H_6_O_3_, C_8_H_8_O_3_, C_8_H_10_O_4_, and C_15_H_12_O_6_, respectively. The peak yielded product ions at *m/z* 451 through heterocyclic ring fission, *m/z* 425 and *m/z* 407 through RDA, and *m/z* 289 through quinone-methide. Compared with previous reports, this compound was identified as (*epi*)catechin dimer [24]. The extract ion chromatogram at *m/z* 441.0827 showed a peak at RT 8.14 min, which produced fragment ions at *m/z* 289 and 169 **(Table 2)** corresponding to the deprotonated ions of (*epi*)catechin and gallic acid, respectively; thus, the compound was identified as (*epi*)catechin-gallate [24]. The extract ion chromatogram at *m/z* 729.1461 showed a peak at RT 6.49 min, which produced a fragment ion at *m/z* 577 through the loss of one galloyl group and fragments at *m/z* 559, 441, and 407 (**Table 2**); thus, the compound was identified as procyanidin-gallate [24]. The extract ion chromatogram at *m/z* 457.0776 showed a peak at RT 5.99 min, producing fragments at *m/z* 305 and 169 (**Table 2**) corresponding to the deprotonated ions of gallocatechin and gallic acid, respectively, hence it was assigned as gallocatechin-gallate [24]. These results showed that the CNC flowers were rich in catechins and their derivatives, which are regarded as effective antioxidants due to their ability to scavenge ROS [27]. In addition, it has been reported that catechin, (*epi*)catechin, catechin dimer, catechin-gallate, procyanidin-gallate, and gallocatechin-gallate are all antioxidants that contribute to beneficial effects on human health [28–31].

The extract ion chromatogram at *m/z* 301.0354 showed a peak at RT 12.17 min, with the fragments at *m/z* 273, 255, 179, and 151 (**Table 2**) corresponding to the loss of CO, CH_2_O_2_, C_7_H_6_O_2_, and C_8_H_6_O_3_, respectively. The compound was identified as quercetin [32]. The extract ion chromatogram at *m/z* 463.08756, 609.1443, and 771.1989 showed peaks at RT 8.50, 12.49, and 6.89 min, respectively. These peaks showed fragments at *m/z* 301 [M-H-162]^-^ (Y_0_^-^), 301 [M-H-146-162]^-^ (Y_0_^-^), and 301 [M-H-162-146-162]^-^ (Y_0_^-^) [33] (**Table 2**), and were thus identified as isoquercitrin [34], rutin [35], and quercetin-glucosyl-rhamnosyl-glucoside [24], respectively. Previous research has shown that quercetin and quercetin glycosides, such as isoquercitrin and rutin, exhibit antioxidant ability in many teas and foods [36–38].

The extract ion chromatogram at *m/z* 285.0405 showed a peak at RT 14.15 min, with fragments at *m/z* 239, 229, 211, and 187 (**Table 2**). The peak was identified as kaempferol [32]. The extract ion chromatogram at *m/z* 447.0933 showed two peaks at RT 9.12 and 9.49 min, producing fragments at *m/z* 285 (Y_0_^-^) [33] (**Table 2**) corresponding to the loss of a galactoside or glucoside and indicative of kaempferol-galactoside (RT 9.12 min) and kaempferol-glucoside (RT 9.49 min) [24]. The extract ion chromatogram at *m/z* 593.1512 and 755.2034 showed peaks at RT 9.16 and 8.12 min, respectively. The peaks displayed fragments at *m/z* 285 [M-H-146-162]^-^ (Y_0_^-^) and 285 [M-H-162-146-162]^-^ (Y_0_^-^) [33] (**Table 2**), consistent with kaempferol-rutinoside [12] and kaempferol-glucosyl-rhamnosyl-glucoside [24, 39], respectively. Earlier studies have shown that kaempferol and kaempferol glycosides are antioxidants because of their abilities to scavenge free radicals [6, 40, 41].

The extract ion chromatogram at *m/z* 479.0831 showed a peak at RT 7.36 min, and fragments at *m/z* 317 (Y_0_^-^) and 316 (Y_0_^—^1) [33] (**Table 2**) corresponding to the loss of 162 and 163 Da, which is consistent with the cleavage of a hexosyl group. The compound was therefore identified as myricitrin-glucoside [24], which is regarded as an antioxidant [42]. The extract ion chromatogram at *m/z* 563.1396 showed a peak at RT 7.31 min and fragments at *m/z* 545, 503, 473, 443, 383, and 353 (**Table 2**). This compound was identified as apigenin-pentosyl-glucoside [43], a flavone glycoside with known antioxidant activity [44]. The extract ion chromatogram at *m/z* 431.0976 showed a peak at RT 8.21 min and fragments at *m/z* 311 and 341 (**Table 2**). This compound was assigned to vitexin [45], which is regarded as a good antioxidant [46].

The extract ion chromatogram at *m/z* 315.05133 showed a peak at RT 14.21 min, and fragments at *m/z* 201 and 229 (**Table 2**). This compound was identified as pollenitin [47], a phenolic compound with good antioxidant activity [48]. The extract ion chromatogram at *m/z* 169.0143 demonstrated a peak at RT 1.42 min. The peak displayed a fragment at *m/z* 125 (**Table 2**) corresponding to the loss of one CO_2_. Thus, it was identified as gallic acid [24], a known antioxidant [49]. The extract ion chromatogram at *m/z* 371.13898 showed a peak at RT 10.43 min and fragments at *m/z* 417 [M-H+HCOOH]^-^ and 209 [M-H-162]^-^ (Y_0_^-^) [33] (**Table 2**); as such, this compound was identified as syringin [50], which plays an antioxidant role in some plants [51].

### Antioxidant activity

Antioxidant activity is influenced by many factors, and a single antioxidant property model cannot fully reflect the antioxidant capacity of all samples [15]. Therefore, more than one antioxidant activity measurement was performed to consider the various mechanisms of antioxidant action. In this study, we carried out three antioxidant models to reflect the antioxidant capacity of CNC flowers: ABTS radical cation scavenging activity, DPPH radical scavenging activity, and ferric reducing antioxidant power (FRAP).

### ABTS radical cation scavenging activity

**Fig 4:**
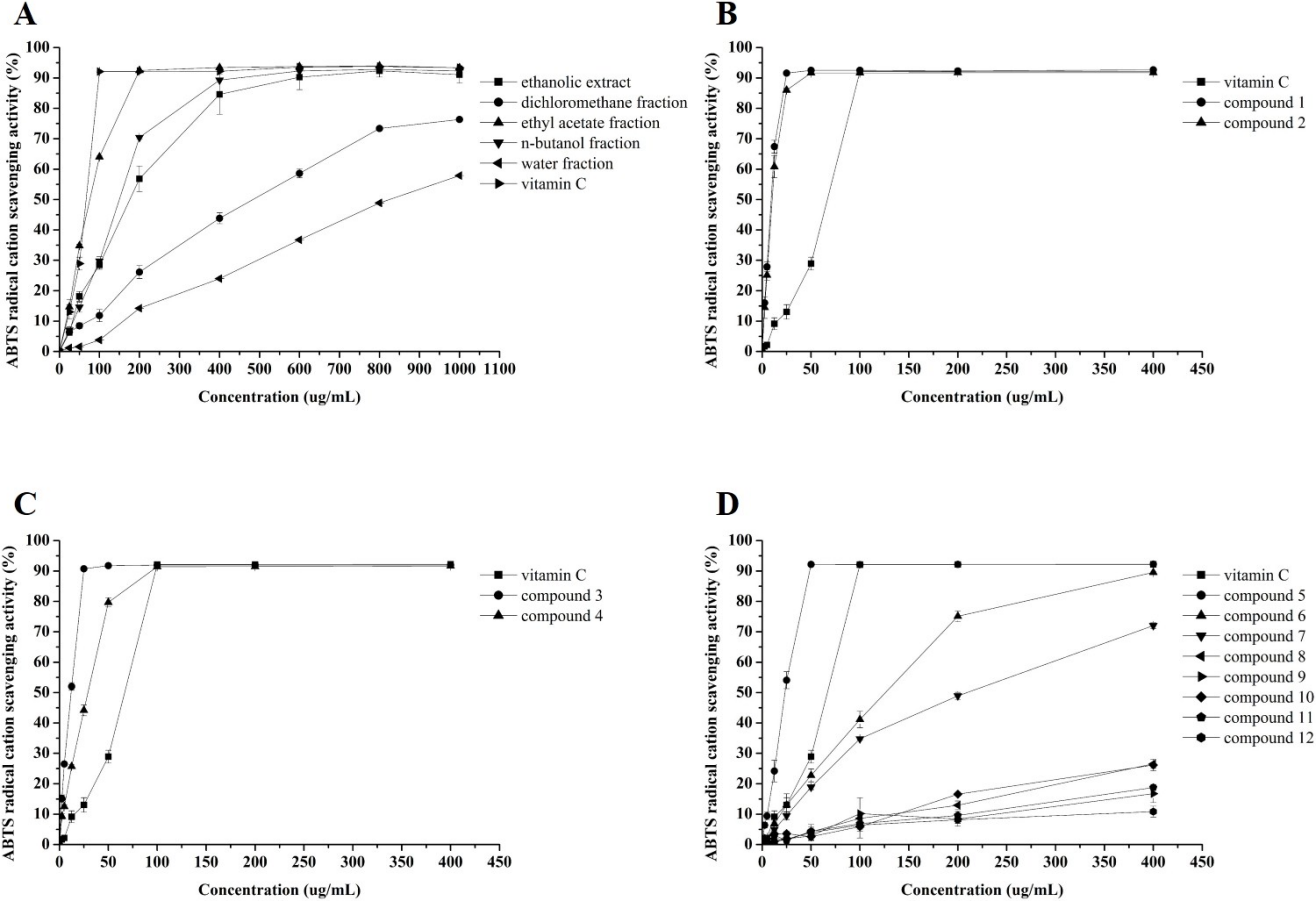
ABTS radical cation scavenging activity of Vc and *C*. *nitidissima* Chi flower fractions (A), compounds **1**–**2** (B), compounds **3**–**4** (C), and compounds **5**–**12** (D). Each value is expressed as mean ± SD (n = 3).

Results showed that all five fractions exhibited scavenging activity for the ABTS radical cation in concentration-dependent manners (**Fig 4A**) and the differences between the fractions were significant (*p* < 0.05). The ethyl acetate fraction, with an EC_50_ of 64.24 ± 1.80 μg/mL (**Table 3**), exhibited significantly higher (*p* < 0.05) ABTS radical cation scavenging activity than that of the other four fractions, the EC_50_ values were 137.40 ± 4.61, 363.90 ± 1.51, and 127.46 ± 5.00 μg/mL, for ethanolic extract dichloromethane and *n*-butanol fraction while the EC_50_ of water fraction was not detected. Interestingly, the trend of the scavenging activity for the ABTS was consistent with the TPC. So the results indicated that the phenolic compounds in the CNC flower played a vital role in scavenging ABTS radical cations.

**Table 3:** 50% effective concentrations (EC_50_) of DPPH and ABTS radical scavenging activities for the 5 fractions and 12 flavonoids isolated from *C*. *nitidissima* Chi flowers.

Samples	DPPH radical scavenging activity (μg/mL)	ABTS radical cation scavenging activity (μg/mL)
Ethanolic extract	142.60 ± 1.46d	137.40 ± 4.61c
Dichloromethane fraction	nd	363.90 ± 1.51a
Ethyl acetate fraction	78.80 ± 0.34e	64.24 ± 1.80f
*n*-butanol fraction	162.60 ± 2.33c	127.46 ± 5.00d
Water fraction	nd	nd
Compound 1	10.36 ± 0.59h	8.22 ± 0.17h
Compound 2	12.68 ± 0.35g	9.70 ± 0.79h
Compound 3	10.41 ± 0.65h	9.86 ± 0.12h
Compound 4	12.89 ± 0.13g	24.15 ± 0.76g
Compound 5	22.40 ± 1.10f	21.56 ± 1.28g
Compound 6	168.62 ± 1.29b	105.33 ± 6.90e
Compound 7	194.85 ± 0.46a	186.16 ± 7.12b
Compound 8	nd	nd
Compound 9	nd	nd
Compound 10	nd	nd
Compound 11	nd	nd
Compound 12	nd	nd

nd, not detected.

EC_50_ was the effective concentration of the test sample that scavenged 50% initial DPPH radical, 50% initial ABTS^+^. The EC_50_ value was obtained by interpolation from linear regression analysis. Values are presented as means ± SD (n = 3), and means in the same column with different lower case letters (a, b, c, d, e, f, g, h) are significantly different (*p* < 0.05).

### DPPH radical scavenging activity

**Fig 5:**
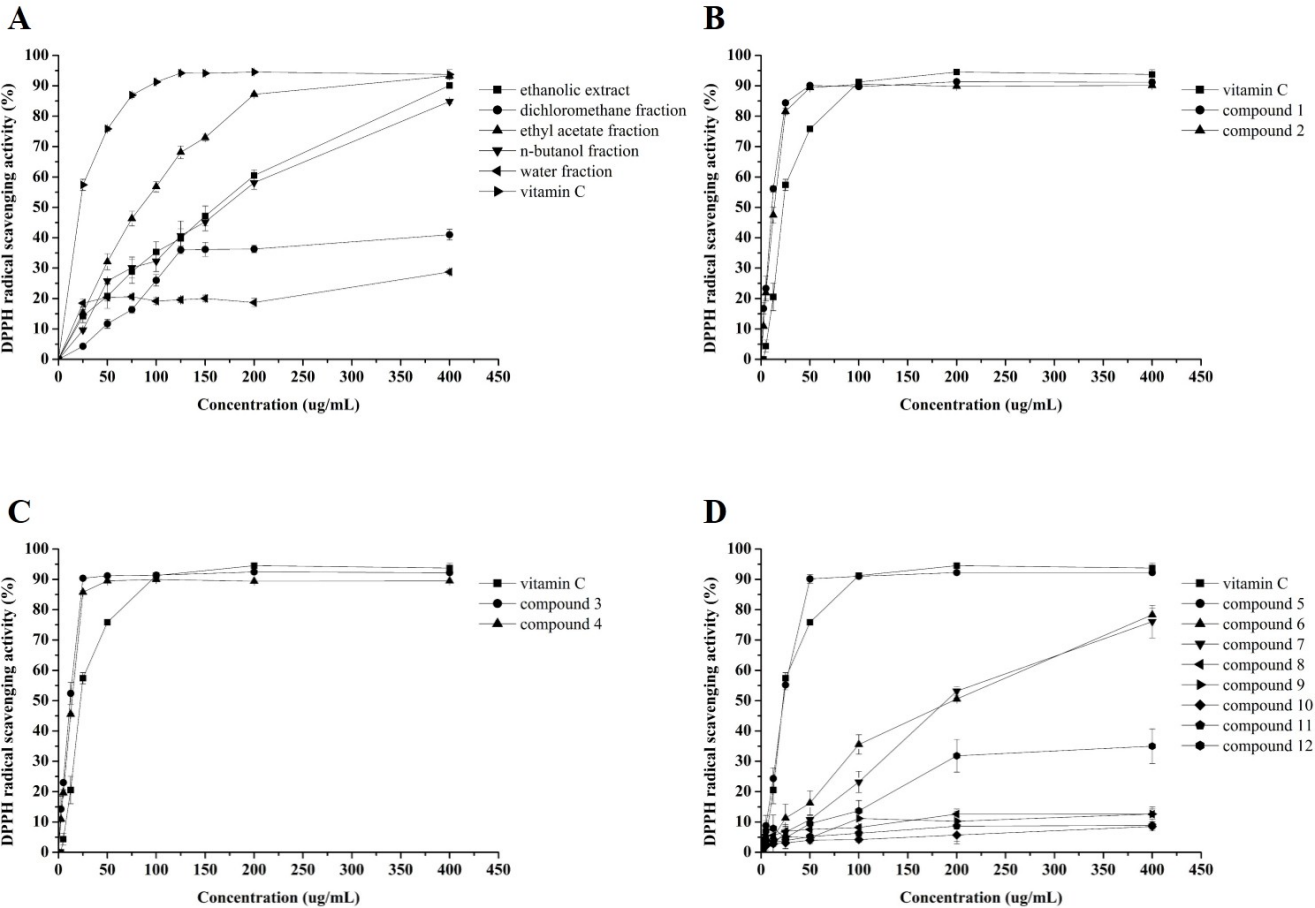
DPPH radical scavenging activity of Vc and *C*. *nitidissima* Chi flower fractions (A), compounds **1**–**2** (B), compounds **3**–**4** (C), and compounds **5**–**12** (D). Each value is expressed as mean ± SD (n = 3).

The DPPH radical scavenging activity results were shown in **Fig 5A**. These results were similar to those of the ABTS radical cation radical scavenging activity. The efficacies were concentration-dependent, and the ethyl acetate fraction showed the highest DPPH radical scavenging activity. The EC_50_ value of the ethyl acetate fraction was 78.80 ± 0.34 μg/mL, and the values of the ethanolic extract and *n*-butanol fraction were 142.60 ± 1.46 and 162.60 ± 2.33 μg/mL, respectively. The EC_50_ values the of dichloromethane and water fractions were not detected. Thus, the order of DPPH radical scavenging activity of the five fractions was ethyl acetate fraction > ethanolic extract > *n*-butanol fraction > dichloromethane fraction > water fraction, and was consistent with the TPC results, which suggested that the phenolic compounds were the main bioactive components in the scavenging of DPPH radicals in the CNC flowers. It has been reported in various studies that higher TPC can lead to significant increases in DPPH radical scavenging activity [8, 13, 52].

### Ferric reducing antioxidant power (FRAP)

**Fig 6:**
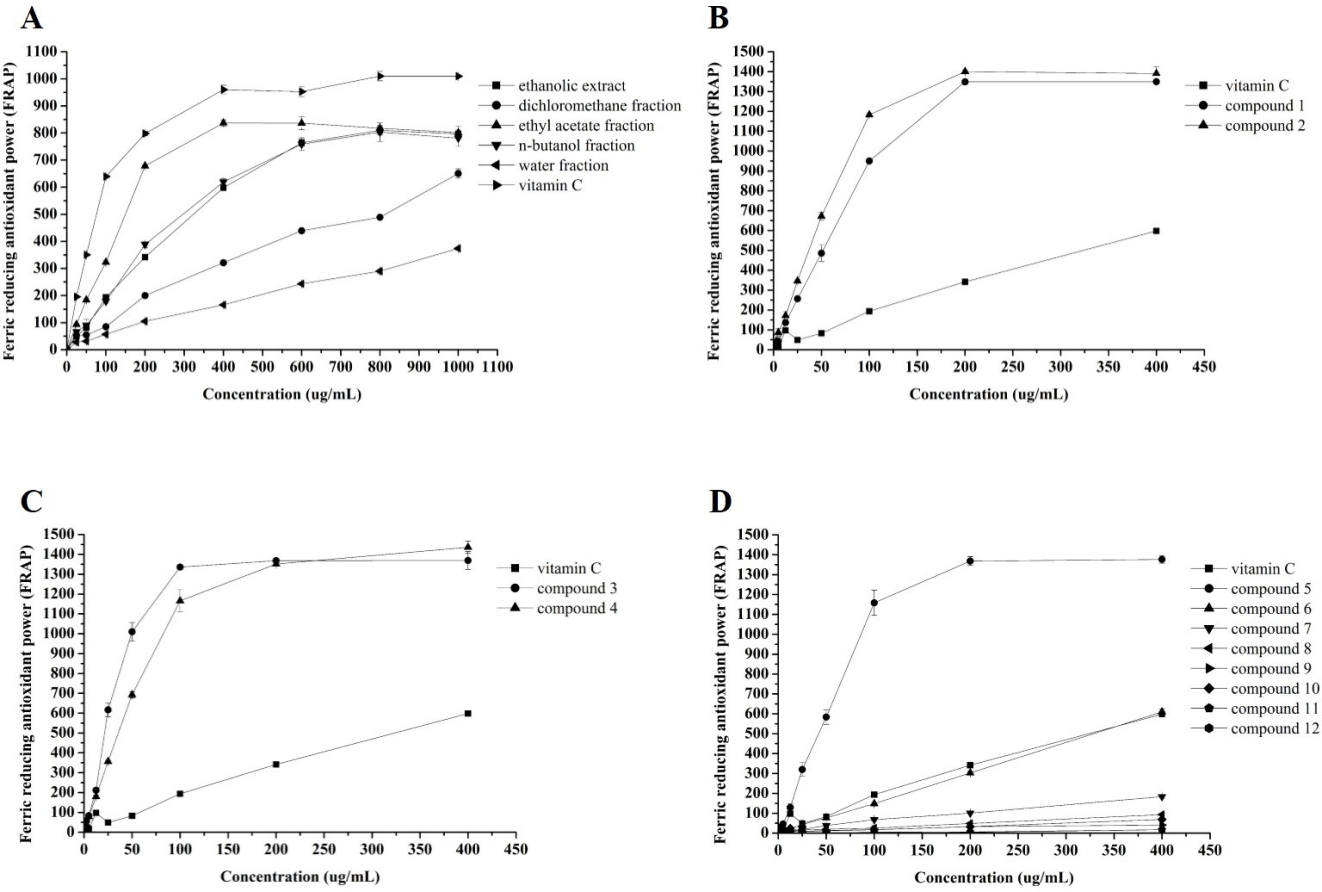
Ferric reducing antioxidant power (FRAP) of Vc and *C*. *nitidissima* Chi flower fractions (A), compounds **1**–**2** (B), compounds **3**–**4** (C), and compounds **5**–**12** (D). Each value is expressed as mean ± SD (n = 3).

Results (**Fig 6A**) showed that the FRAP of the CNC flower fractions increased with their dosage, which was consistent with the ABTS^+^ and DPPH radical scavenging activity. Among the five fractions, the ethyl acetate and water fractions showed the highest and lowest FRAP, respectively, at concentrations ranging from 25 μg/mL to 1,000 μg/mL and FRAP values ranging from 93.49 ± 1.71 to 801.49 ± 2.30 and 27.93 ± 3.53 to 374.04 ± 7.04 μM FeSO_4_, respectively. The values of the *n*-butanol fraction ranged from 66.93 ± 1.33 to 780.60 ± 3.11 μM FeSO_4_, similar to that of the ethanolic extract (from 49.15 ± 1.39 to 796.60 ± 7.26 μM FeSO_4_), but higher than that of the dichloromethane fraction (from 52.48 ± 1.84 to 650.93 ± 1.69 μM FeSO_4_). The results showed that TPC played the major role in the FRAP of the CNC flowers. Our results are supported by previous studies suggesting that phenolic compounds might be responsible for a large proportion of the antioxidant activity determined by FRAP assay in some plants [53, 54].

### Correlation analysis between antioxidant capacity and the total phenolic content (TPC)

Previous studies have reported that the higher phenolic content in the extracts resulted in a higher antioxidant activity, which was in agreement with a positive correlation between TPC and antioxidant activity [1, 15]. So in order to obtain the detail correlations between antioxidant capacity and the TPC of the five fractions, the correlation analyses were conducted. As shown in **Table 4**, the significant correlations (*p* < 0.01) between the antioxidant properties and TPC of the five fractions were found. The TPC was highly associated with scavenging ability against ABTS (r = 0.890, 0.983, 0.745, 0.859, and 0.992, for ethanolic extract, dichloromethane fraction, ethyl acetate fraction, *n*-butanol fraction, and water fraction, respectively), DPPH (r = 0.979, 0.897, 0.893, 0.973, and 0.694, for the five fractions, respectively) and FRAP (r = 0.946, 0.991, 0.823, 0.933, and 0.995, for the five fractions, respectively). So the data indicated that the phenolic compounds in the five fractions of CNC flowers were considered responsible for effective antioxidant properties.

**Table 4:** Pearson’s correlation coefficients for total phenolic content (TPC) of the five fractions of *C*. *nitidissima* Chi flowers and antioxidant activity.

Ethanolic extract		TPC	ABTS	DPPH	FRAP
	TPC	1	0.890**	0.979**	0.946**
	ABTS	.	1	0.991**	0.980**
	DPPH			1	0.990**
	FRAP				1
Dichloromethane fraction		TPC	ABTS	DPPH	FRAP
	TPC	1	0.983**	0.897**	0.991**
	ABTS	.	1	0.907**	0.987**
	DPPH			1	0.911**
	FRAP				1
Ethyl acetate fraction		TPC	ABTS	DPPH	FRAP
	TPC	1	0.745**	0.893**	0.823**
	ABTS	.	1	0.997**	0.969**
	DPPH			1	0.976**
	FRAP				1
*n*-Butanol fraction		TPC	ABTS	DPPH	FRAP
	TPC	1	0.859**	0.973**	0.933**
	ABTS		1	0.983**	0.973**
	DPPH			1	0.983**
	FRAP				1
Water fraction		TPC	ABTS	DPPH	FRAP
	TPC	1	0.992**	0.694**	0.995**
	ABTS		1	0.650**	0.994**
	DPPH			1	0.746**
	FRAP				1

** Correlation is significant at the 0.01 level (2-tailed).

### Principal component analysis (PCA)

**Fig 7:**
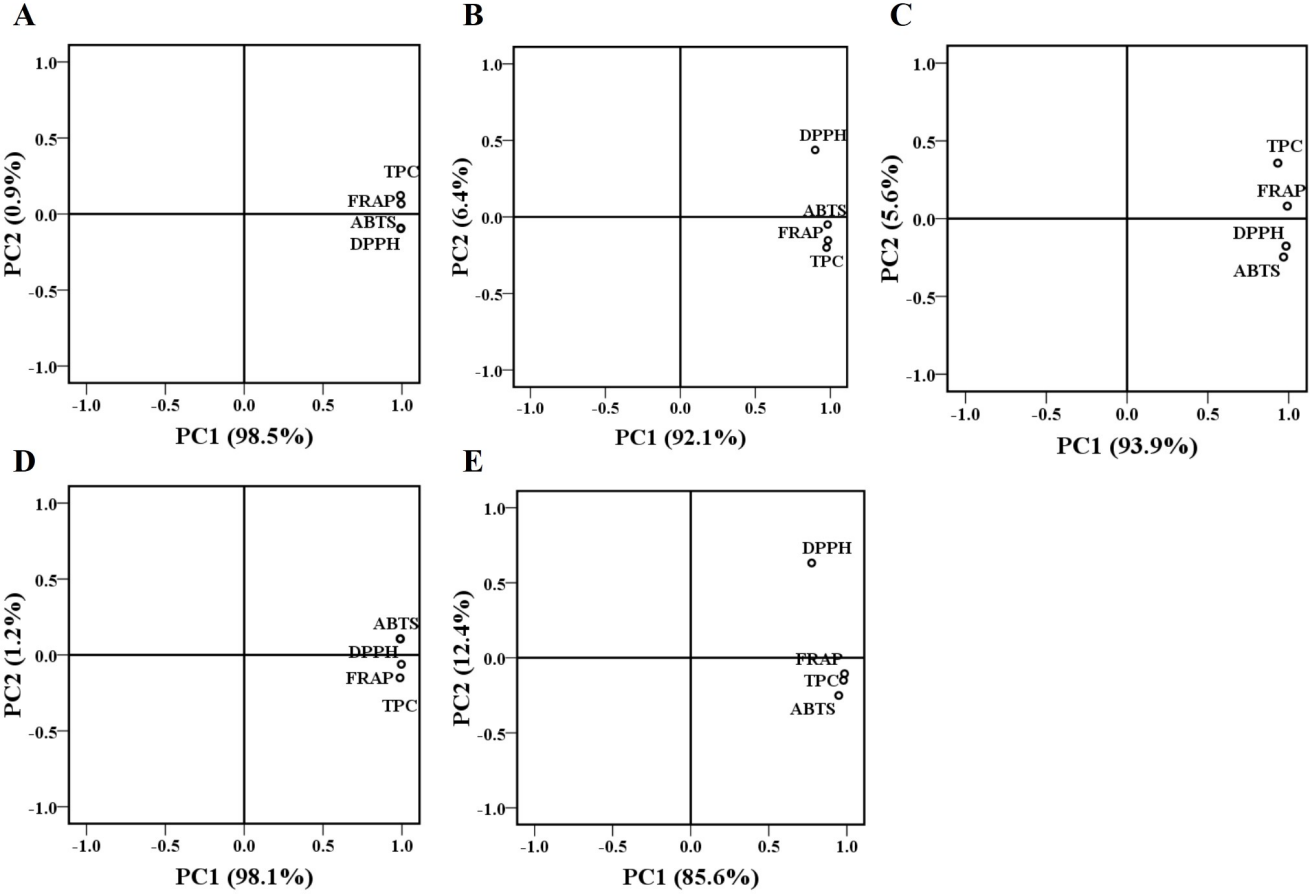
Principal component analysis (PCA) loading plot of total phenolic content (TPC) and antioxidant activity of the ethanolic extract (A) dichloromethane fraction (B), ethyl acetate fraction (C), *n*-butanol fraction (D), and water fraction (E) of *C*. *nitidissima* Chi flowers.

To investigate the interrelationships between the different variables and to find the optimum number of extracted principal components, principal component analysis (PCA) was applied to reduce the original variables (TPC, ABTS, DPPH, and FRAP) in a smaller number of underlying variables (principal component) [15]. The principal component analysis (PCA) and their correlations were shown in **Fig 7**and **Table 5**. Two principal components together of the ethanolic extract, dichloromethane fraction, ethyl acetate fraction, *n*-butanol fraction, and water fraction were 99.4%, 98.5%, 99.5%, 99.3%, and 98.0%, respectively. The first principal component (PC1) correlated well with TPC, ABTS, DPPH, and FRAP. In addition, TPC, ABTS, DPPH, and FRAP were significantly correlated with each other in the five fractions of CNC flowers. So the strong correlations among TPC, ABTS, DPPH, and FRAP suggested that the contents of phenolic compounds and antioxidant properties were highly correlated with each other.

**Table 5:** Component matrix of the five fractions from *C*. *nitidissima* Chi flowers.

	Ethanolic extract		Dichloromethane fraction		Ethyl acetate fraction		*n*-Butanol fraction		Water fraction	
	Component									
	1	2	1	2	1	2	1	2	1	2
TPC	0.991	0.122	0.974	-0.200	0.933	0.357	0.988	-0.151	0.978	-0.150
ABTS	0.993	-0.096	0.981	-0.049	0.968	-0.247	0.990	0.105	0.948	-0.251
DPPH	0.993	-0.092	0.898	0.438	0.984	-0.176	0.989	0.107	0.775	0.632
FRAP	0.994	0.067	0.983	-0.152	0.992	0.080	0.996	-0.062	0.985	-0.106

Extraction method: Principal component analysis.

Two components extracted.

### Antioxidant activity of 12 flavonoids and their potential structure-activity relationship

To evaluate the antioxidant capacity of 12 flavonoids isolated from CNC flowers, ABTS radical cation scavenging activity, DPPH radical scavenging activity, and ferric reducing antioxidant power (FRAP) analyses were conducted. As seen in **Figs 4**–**6**, significant differences in antioxidant capacity were investigated among the 12 flavonoids.

As shown in **Table 3**, the EC_50_ values of compounds **1** and **3** were significantly lower than that of compound **5** in DPPH and ABTS^+^ radical scavenging activities, with the FRAP results similar to the other two models. This indicated that the antioxidant capacity of flavonoids of different classes was positively correlated with the number of hydroxyl groups. From further analysis, catechin and quercetin both possess the *o*-catechol group in the B-ring, whereas kaempferol only possesses one hydroxyl group in the B-ring; thus, the *o*-catechol group is considered the major group for the antioxidant capacity. In addition, the results proved that 2,3-double bond in conjugation with the 4-oxo group in the C ring is not a determinant structural feature for the antioxidant capacity of flavonoids [55].

The antioxidant capacity results of compounds **1** and **2** (**Figs 4B**, **5B** and **6B**) showed that although compound **2** had more hydroxyl groups, both compounds had the same antioxidant capacity because compound **2** had a large substituent with steric hindrance that reduced activity [56, 57]. As seen in **Figs 4C**, **5C** and **6C**, there were significant differences (*p* < 0.05) between the antioxidant capacity of compounds **3** and **4**. The EC_50_ values of the DPPH and ABTS^+^ radical scavenging activities of compound **3** were significantly (*p* < 0.05) lower than those of compound **4** (**Table 3**). Similarly, the antioxidant capacity of compound **5** was significantly (*p* < 0.05) stronger than that of compounds **6**–**12** (**Figs 4D**, **5D** and **6D)**, which were all kaempferol glycosides, with the antioxidant capacity decreasing with increasing number of glycosides (**Table 3**). Our results indicated that glycosides could reduce the antioxidant capacity of flavonoids, such as quercetin and kaempferol, due to their production of steric hindrance [56].

## Conclusions

*C*. *nitidissima* Chi flowers, regarded as a medicinal and edible plant in China, showed strong antioxidant capacity. All five fractions of the CNC flowers demonstrated the activity to scavenge ABTS^+^, DPPH radicals, and ferric reducing antioxidant power (FRAP), especially the ethyl acetate fraction. Pearson’s correlation and PCA of the TPC of the five fractions and antioxidant capacity indicated that the phenolic compounds were the major antioxidants in CNC flowers. In total, 21 phenolic compounds, all of which are antioxidants, were identified by HPLC Triple TOF MS/MS analysis in the ethanolic extract of CNC flowers. In addition, 12 flavonoid compounds were isolated from CNC flowers, and the potential structure-activity relationship among the 12 compounds showed that (1) the *o*-catechol group in the B-ring played an important role in the antioxidant capacity of the flavonoids and (2) steric hindrance, produced by glycosides and other groups, could reduce the antioxidant capacity of the flavonoids.

## Supporting information

S1 Fig MS/MS spectra of identified compounds. (DOCX)
