# Supplementary figures and images for: Antioxidant capacity of phenolics in Camellia nitidissima Chi flowers and their identification by HPLC Triple TOF MS/MS
Source: PLoS One. 2018 Apr 10;13(4):e0195508. doi: 10.1371/journal.pone.0195508 (PMC5892910; doi:10.1371/journal.pone.0195508)

**Supplementary data**

**Figure S1. MS/MS spectra of identified compounds**


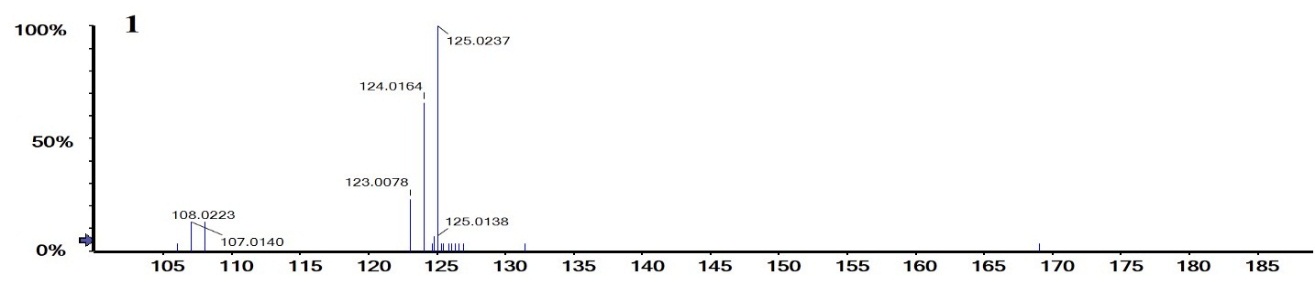

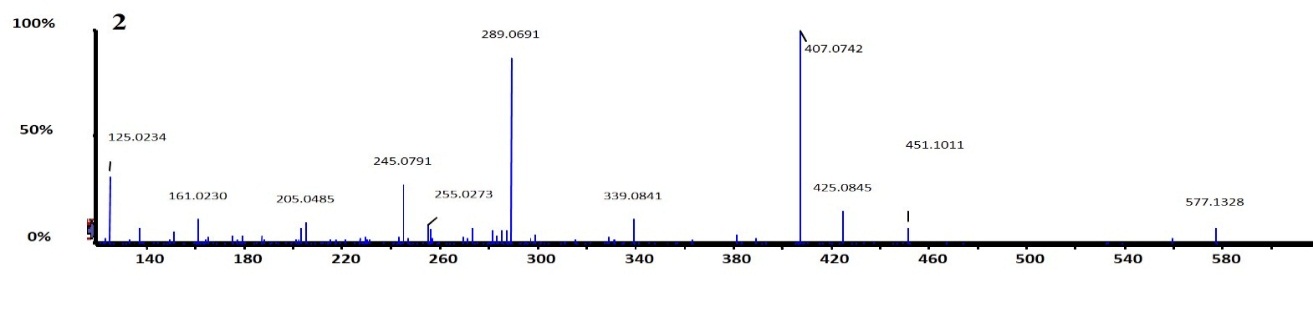

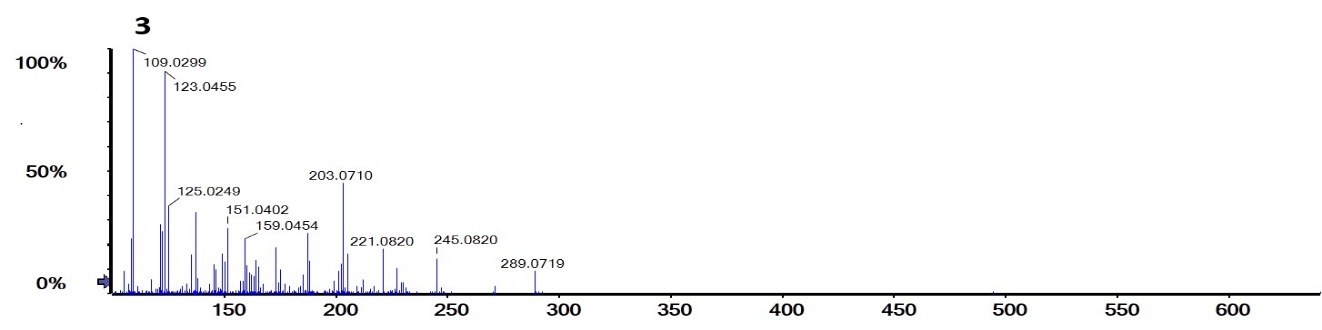

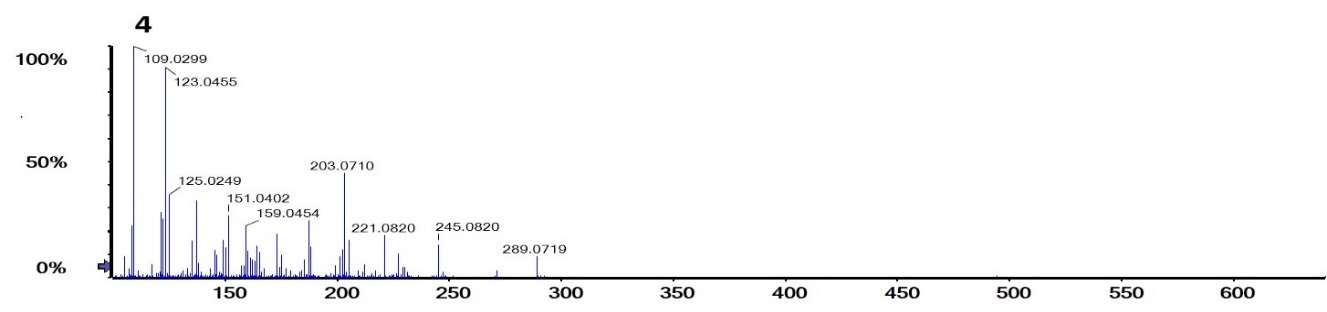

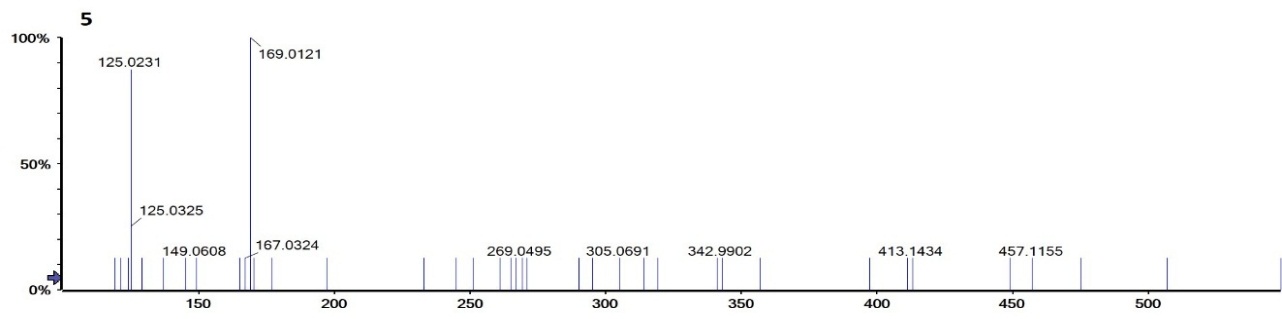

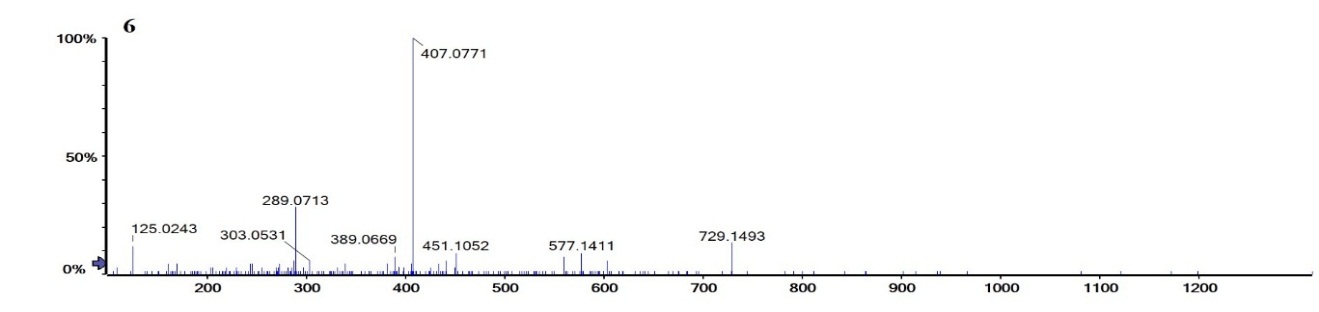

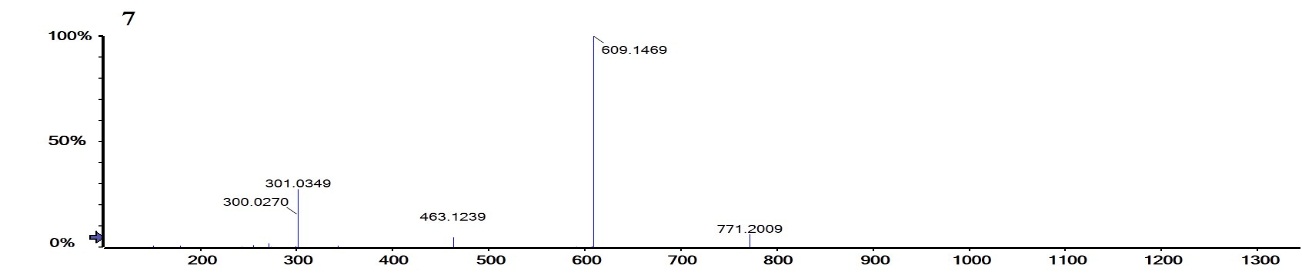

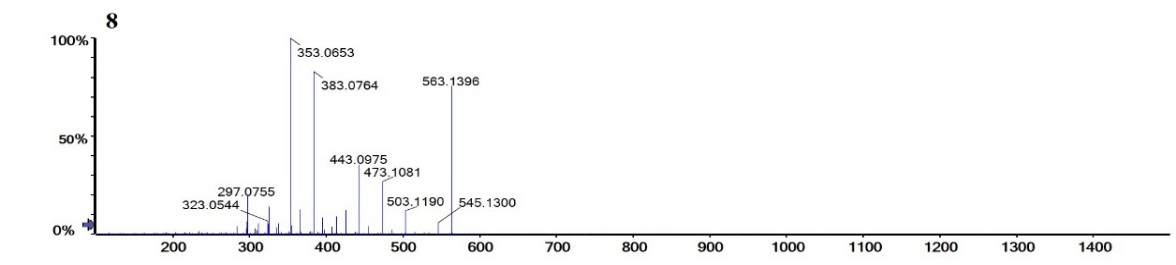

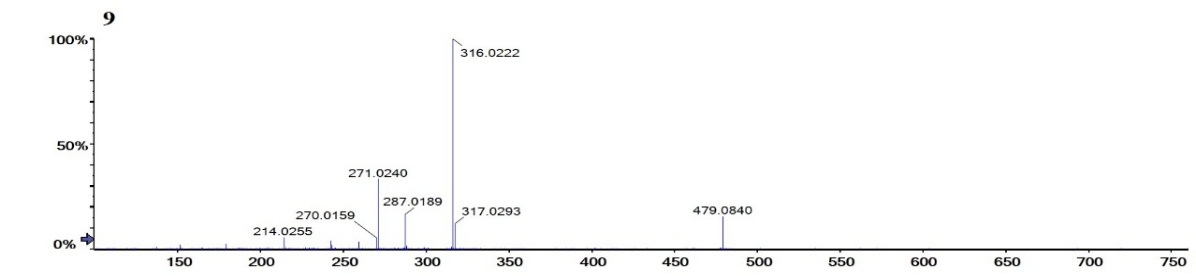

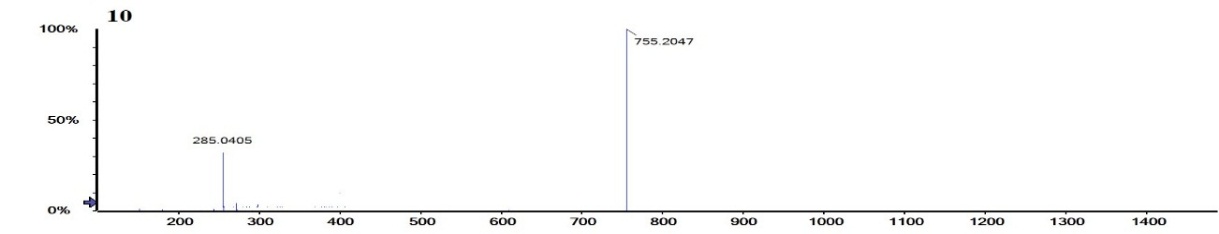

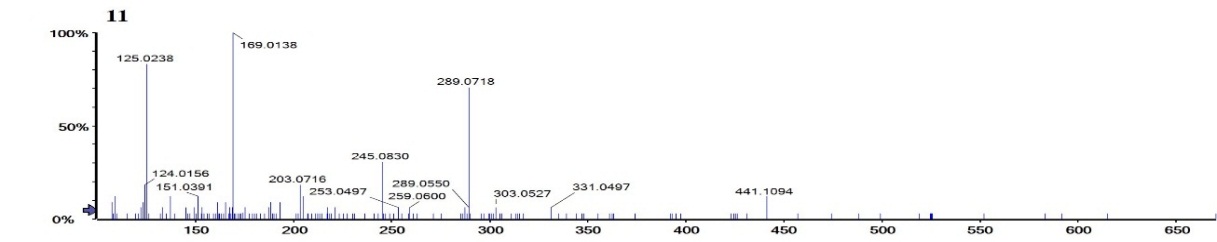

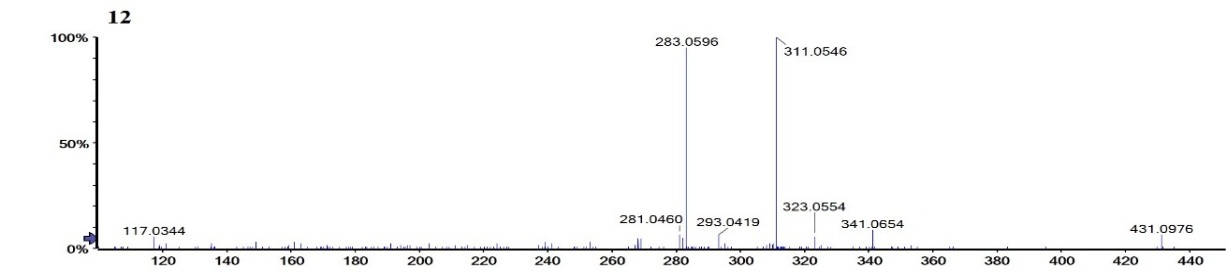

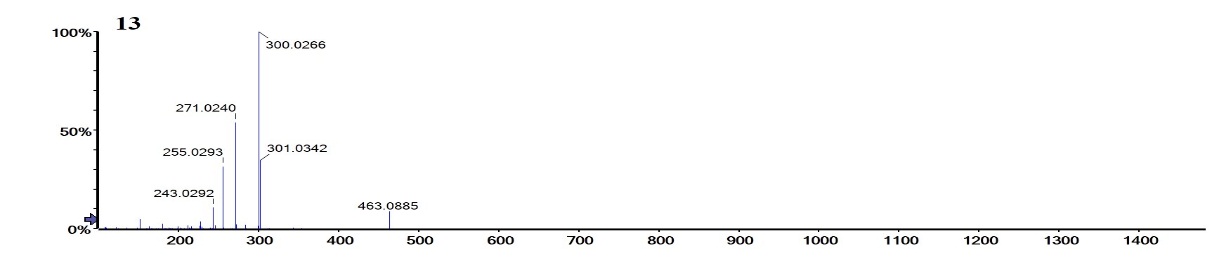

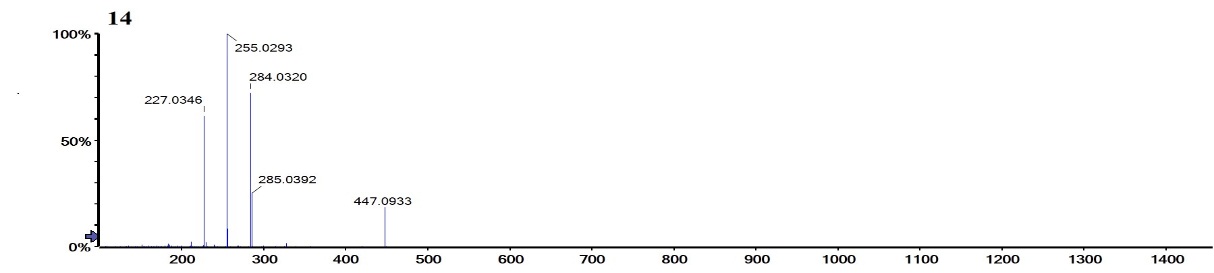

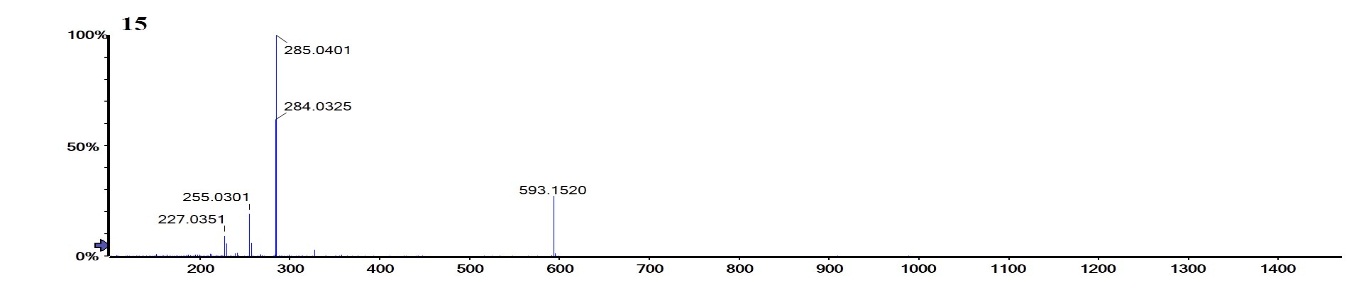

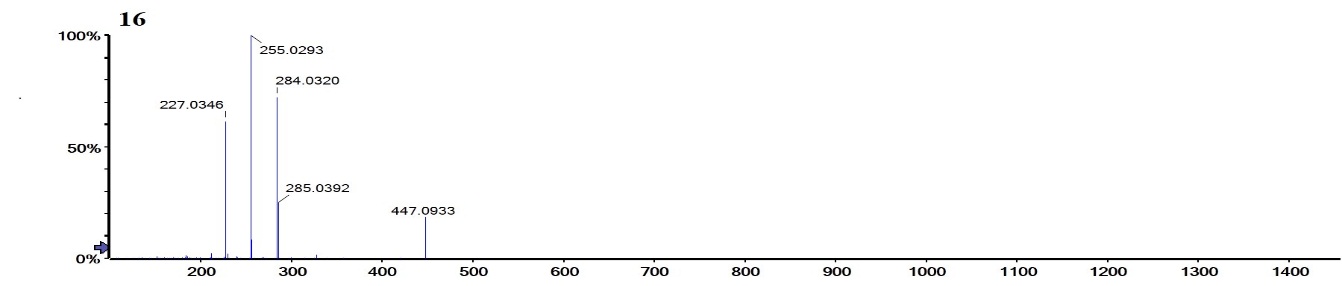

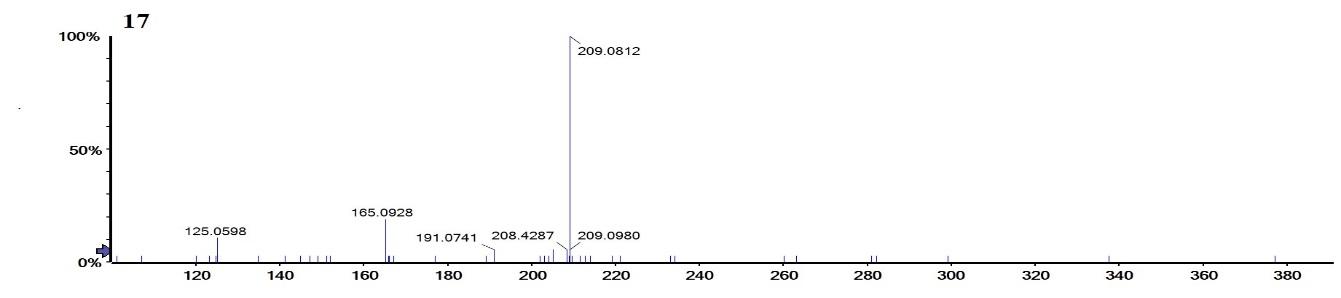

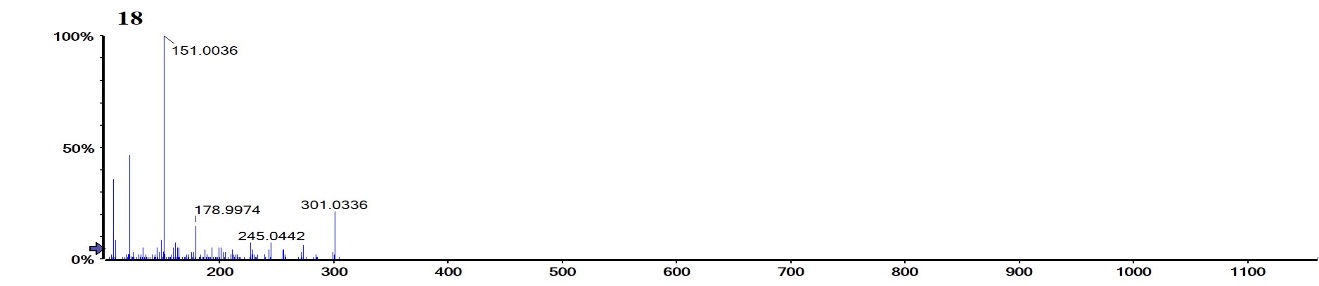

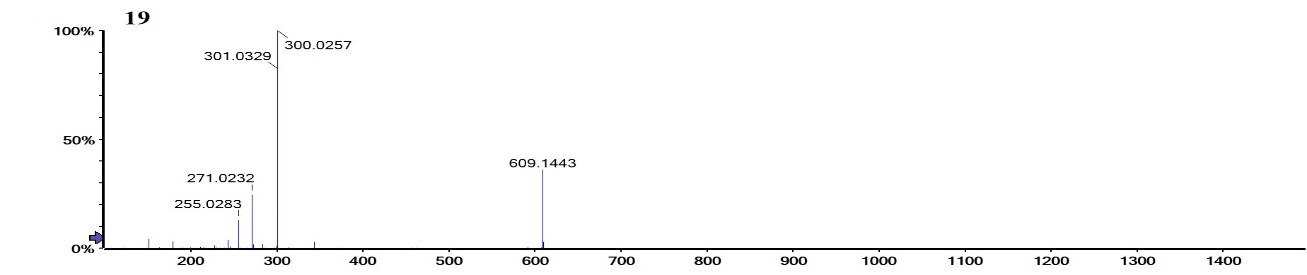

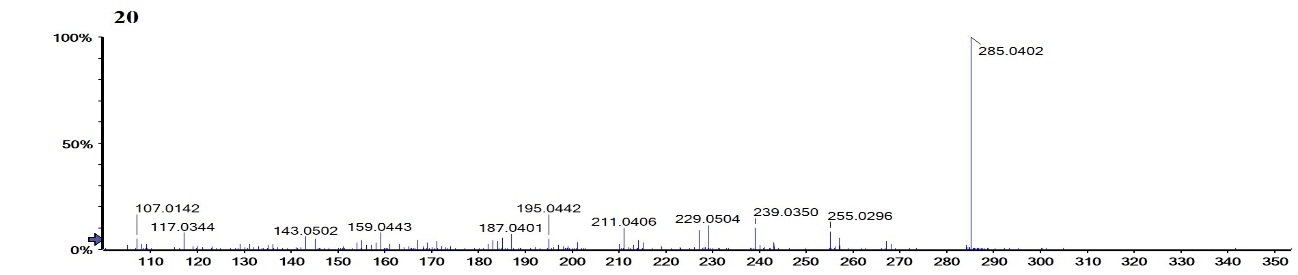

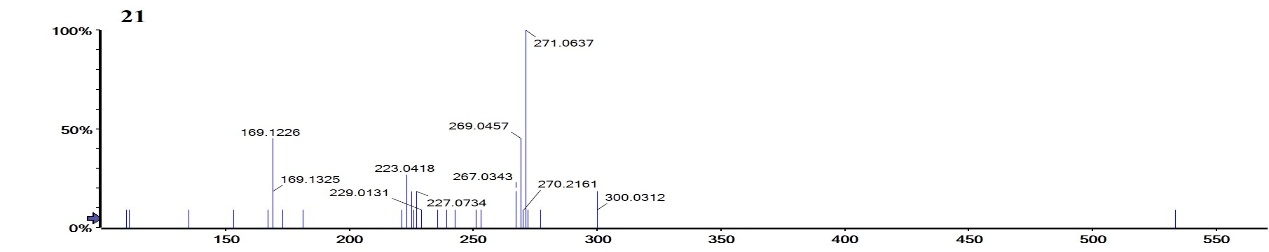

Supplement: S1 Fig — (DOCX) [file pone.0195508.s001.docx]
